# Critical evaluation of the role of external calibration strategies for IM-MS

**DOI:** 10.1007/s00216-022-04263-5

**Published:** 2022-08-12

**Authors:** Max L. Feuerstein, Maykel Hernández-Mesa, Younes Valadbeigi, Bruno Le Bizec, Stephan Hann, Gaud Dervilly, Tim Causon

**Affiliations:** 1 Department of Chemistry, Institute of Analytical Chemistry, University of Natural Resources and Life Sciences, Muthgasse 18, 1190 Vienna, Austria; 2 Oniris, INRAE, LABERCA, 44300 Nantes, France

**Keywords:** Ion mobility-mass spectrometry, DFT, CCS, Stable isotope labelling, Steroids

## Abstract

The major benefits of integrating ion mobility (IM) into LC–MS methods for small molecules are the additional separation dimension and especially the use of IM-derived collision cross sections (*CCS*) as an additional ion-specific identification parameter. Several large *CCS* databases are now available, but outliers in experimental interplatform IM-MS comparisons are identified as a critical issue for routine use of *CCS* databases for identity confirmation. We postulate that different routine external calibration strategies applied for traveling wave (TWIM-MS) in comparison to drift tube (DTIM-MS) and trapped ion mobility (TIM-MS) instruments is a critical factor affecting interplatform comparability. In this study, different external calibration approaches for IM-MS were experimentally evaluated for 87 steroids, for which ^*TW*^*CCS*_*N2*_, ^*DT*^*CCS*_*N2*_ and ^*TIM*^*CCS*_*N2*_ are available. New reference *CCS*_*N2*_ values for commercially available and class-specific calibrant sets were established using DTIM-MS and the benefit of using consolidated reference values on comparability of *CCS*_*N2*_ values assessed. Furthermore, use of a new internal correction strategy based on stable isotope labelled (SIL) internal standards was shown to have potential for reducing systematic error in routine methods. After reducing bias for *CCS*_*N2*_ between different platforms using new reference values (95% of ^*TW*^*CCS*_*N2*_ values fell within 1.29% of ^*DT*^*CCS*_*N2*_ and 1.12% of ^*TIM*^*CCS*_*N2*_ values, respectively), remaining outliers could be confidently classified and further studied using DFT calculations and *CCS*_*N2*_ predictions. Despite large uncertainties for in silico *CCS*_*N2*_ predictions, discrepancies in observed *CCS*_*N2*_ values across different IM-MS platforms as well as non-uniform arrival time distributions could be partly rationalized.

**Supplementary Information:**

The online version contains supplementary material available at 10.1007/s00216-022-04263-5.

## Introduction

High-resolution mass spectrometry (MS) coupled to liquid chromatography (LC) has evolved as key technology for the analysis of small molecules in metabolomics, lipidomics, environmental analytics and related disciplines [1–3]. Due to the chemical diversity of small molecules and the large variety of possible isomers and isobars present, increasing method selectivity by enhancing peak capacity remains of great interest [4, 5]. In this regard, ion mobility coupled to mass spectrometry (IM-MS) increases peak capacity and improves signal-to-noise ratios for such applications [2, 3, 6, 7]. Importantly, due to the speed of gas-phase separations of ions, IM is readily integrated in LC–MS workflows without compromising total analysis time [8, 9]. Different types of IM-MS analysers are now commercially available including drift tube (DTIM-MS), travelling wave (TWIM-MS) or trapped ion mobility coupled to MS detectors (TIM-MS) [8, 10]. In IM-MS, analyte ions are separated based on opposing forces of an applied electric field and collisions with a neutral buffer gas (typically nitrogen) before entering the MS analyser [8, 11]. As a derived property, the collision cross section (*CCS*) of an ion can be calculated with excellent interlaboratory precisions of typically in the range of 1–2% reported in several studies [12–14]. Moreover, the increasing number of curated and freely available *CCS* databases [15–17] has popularized the use of *CCS* values as an identification parameter intended for standard-free identification workflows [17–19].

However, in contrast to mass to charge ratios (*m*/*z*), *CCS* is a conditional value of an ion that cannot be calculated in a straightforward manner. Moreover, experimentally observed ion structures may be influenced by the employed experimental parameters including the ESI source conditions, as well as applied voltages and source temperatures [11]. Of the major commercial instrument types, DTIM-MS is most closely related to fundamental IM theory, and ^*DT*^*CCS*_*N2*_ values can be derived using a primary method of measurement (i.e. stepped-field method) or via secondary methods (i.e. single-field calibrated) on a routine basis [12]. However, uncertainties associated with reference values remain due to lack of standardization and reference materials. These uncertainties therefore directly influence the different secondary calibration approaches that are applied on a routine basis for *CCS* determination using DTIM-MS, TWIM-MS and TIM-MS [20]. Especially for TWIM-MS, the applied calibration strategy including the selection of calibrant ions has been reported to influence comparability of ^*TW*^*CCS*_*N2*_ values [21]. Fundamental differences between ^*TW*^*CCS*_*N2*_ and ^*DT*^*CCS*_*N2*_ values due to ion transport and ion heating effects have also been discussed as potential sources of differences observed between IM-MS platforms [22].

In context of small molecules, steroid analysis is of special interest due to the large number of possible isomers, and benefits of IM-MS for steroid analysis were demonstrated previously [7, 13, 15, 23, 24]. From the analytical applications perspective, the comparability of *CCS*_*N2*_ for steroid analysis using three different IM-MS technologies was recently investigated, and interlaboratory bias of < 2% for the majority of the investigated ions was demonstrated [14]. Nevertheless, large deviations (up to 7%) of CCS values derived from TWIM-MS and TIM-MS to ^*DT*^*CCS*_*N2*_ values have also been reported [14, 25]. In addition to the possibility of fundamental differences in ion conformations generated and sampled by the different IM-MS instruments, systematic bias of ^*TW*^*CCS*_*N2*_ values compared to other IM-MS instruments is evident and may have its origin in the applied external calibration [14, 25]. Alongside analytically challenging examples such as ions with complex arrival time distributions and the high level of effort required for computational prediction of *CCS* values using density functional theory (DFT) for large datasets [26], this issue leaves the use of *CCS*_*N2*_ values as an IM-MS technology-independent identification parameter in a currently unsatisfactory position [14]. Therefore, to further investigate the effect of the applied calibration approach and especially the role of reference values used for ^*TW*^*CCS*_*N2*_ calibration, alternative external calibration and internal correction approaches are explored in the present work. In addition to matching calibrant class to sample type, unified calibrant sets and stable isotope label (SIL) internal correction strategies are investigated. With a goal of elucidating the magnitude of calibration-dependent bias, this work aims to support efforts toward long-term applicability of *CCS*_*N2*_ values for analytical small molecule applications.

## Materials and methods

### Chemicals and reagents

Steroid standards were purchased from Steraloids (Newport, RI, USA), Sigma-Aldrich (St. Louis, MO, USA) and National Measurement Institute (NMI, Pymble, Australia). Stock solution (100 μg/mL or 1 mg/mL) were stored in ethanol at − 20 °C. Mixed solutions (10 µg/mL) of these steroids were prepared for LC-IM-MS analysis as described elsewhere [14].

Ultrapure water from a Milli-Q IQ 7000 purification system and LC-Pak® polisher cartridge (Merck Chemicals and Life Science GmbH, Vienna) along with LC–MS grade acetonitrile (ACN) and formic acid (FA) from Sigma-Aldrich were used to prepare eluents and to dilute standards prior to LC-DTIM-MS analysis. ESI-L Tune Mix (G1969-85,000, Agilent Technologies) along with 0.1 mmol/L HP-0321 (Agilent Biopolymer Reference Kit) were used for mass calibration of the Agilent 6560 DTIM-QTOF and for determination of ^*DT*^*CCS*_*N2*_ values using the single-field calibration method [12] and was tested for ^*TW*^*CCS*_*N2*_ calibration on the Waters Synapt G2-S.

Sodium formate (0.5 mmol/L in 90:10 (v/v) 2-propanol:water was prepared from sodium hydroxide (1 mol/L, Fisher Chemical™) and formic acid (Promochem®) supplied by Fisher Scientific (Loughborough, UK) and was used for mass calibration of the Synapt G2-S. Major Mix IMS/ToF Calibration Kit (Waters, Wilmslow, UK) was used for ^*TW*^*CCS*_*N2*_ calibration and is referred to as “CCS Major Mix” in the following sections.

Leucine enkephalin (Waters) prepared in (50:50 (v/v) 0.2% FA:ACN) was used for lock mass correction. For LC-TWIM-MS analysis, ACN and 2-propanol (LC–MS Chromasolv® grade) were obtained from Sigma-Aldrich (St. Louis, Mo, USA), water (HiperSolv Chromanorm® for HPLC) was provided by VWR International (West Chester, PA, USA) and FA used to acidify the mobile phases was purchased from LGC Standards GmbH (Wesel, Germany).

An Agilent 6560 IM-QTOFMS equipped with a Dual Jetstream ESI source was used for determination of new ^*DT*^*CCS*_*N2*_ reference values for Waters CCS Major Mix and stable isotope labelled (SIL) steroids (see Electronic Supplementary Information Tables S1–S3).

### Sample preparation

Agilent ESI-L tune mix was prepared according to manufacturer instructions for the ion source used in this study. Briefly, a 1:10 dilution of ESI-L Tune Mix was prepared in 95:5 (v/v) water:ACN and additionally spiked with HP-0321 (hexamethoxyphosphazine). A set of 87 steroids used in previous interlaboratory comparisons of different IM-MS systems was also used in this study [13, 14]. Mixtures of standards were prepared at 0.5 µg/mL for LC-TWIM-MS analysis; water-soluble steroids were prepared in 95:5 (v/v) 0.1% FA:ACN, while hydrophobic steroids (e.g. sterol esters) were prepared in 50:50 (v/v) 0.1% FA:ACN according to an established protocol [14]. For investigating the possibility of SIL-supported internal correction for ^*TW*^*CCS*_*N2*_ calibration, standard mixtures were spiked with SIL-steroid standards to yield a final concentration of 0.5 mg/L.

### Instrumentation and data acquisition

Previously established RPLC and DTIM-MS methods were used to analyse stable isotope labelled steroids and CCS Major Mix calibrant ions. For this purpose, an Agilent 6560 IM-QTOFMS equipped with a Dual Jetstream ESI source was used. For DTIM-MS analysis, mixtures were directly infused using a syringe pump (KD Scientific, series 100, USA) at a flow rate of 20 µL/min. Applied method parameters have been previously reported and are summarized in the Electronic Supplementary Information [14].

The same LC method was used for front-end separation along with TWIM-MS measurements using a Waters Synapt G2-S TWIM-MS system. An Acquity UPLC System (Waters) equipped with an Acquity UPLC®(BEH C18, 2.1 mm × 100 mm, 1.7 mm; Waters) was used along with previously established methods [15]. Prior to analysis, sodium formate (0.5 mmol/L in 90:10 (v/v) 2-propanol:water was used for mass calibration, while lock mass correction was applied during measurements using leucine enkephalin (1–2 ng/mL in 50:50 (v/v) 0.2% FA:ACN). The instrument was *CCS* calibrated using (1) CCS Major Mix or using alternative calibration approaches with (2) Agilent ESI-L tune mix or (3) a mix of steroids with newly established *CCS*_*N2,ref*_ values (see Electronic Supplementary Information Table S3). The two commercial solutions were prepared according to vendor instructions, while the mixture of steroid standards was prepared at a concentration of 10 mg/L in 50:50 0.1% (v/v) FA:ACN. The TWIM-MS acquisition methods in ESI^+^ and ESI^−^ modes were optimized according to the applied *CCS* calibration mixture (see Electronic Supplementary Information). Finally, ^*TIM*^*CCS*_*N2*_ data from a recent study was also used for comparisons of new IM-MS calibration and correction strategies [14].

### Data processing and visualization

Single-field calibration for DTIM-MS was applied using Agilent IM-MS Browser 10.0. Single-field calibrated data was demultiplexed and pre-processed using PNNL Preprocessor 3.0 (2021.04.21) [27], and Agilent MassHunter Mass Profiler 10.0 was used for peak picking and alignment of triplicate measurements [14].

For TWIM-MS, DriftScope V.2.8 included in MassLynx 4.2 software (Waters) was used to determine the ^*TW*^*CCS*_*N2*_ calibration functions, which were saved into corresponding measurement data files. Individual data files were investigated using DriftScope and MS-DIAL 4.60 [28, 29] was used to batch-process TWIM-MS data. To this end, datafiles in raw format were converted to.ibf files using the built-in converter. Settings used for peak picking and alignment are provided in the Electronic Supplementary information. ^*TW*^*CCS*_*N2*_ values were calculated from arrival times using the Enhanced Duty Cycle (EDC) coefficient to correct arrival times [30], and a detailed description of the applied calibration approach is presented in the Electronic Supplementary Information.

Finally, Microsoft® Office® (Excel® and PowerPoint®) and R (4.1.2) [31] together with RStudio (2021.9.1.372) [32] were used for data analysis, visualization and creation of final figures.

### Stable isotope label (SIL)-based internal correction

For establishing an application-specific internal correction strategy, linear models to describe the relationship between the *CCS*-ratio and modified *CCS* (*CCS’* = ^*DT*^*CCS*_*N2,ref*_* ∙√(µ)⁄z* using reduced mass *µ* and the charge number *z*) were established based on a set of twelve SIL steroids, for which new ^*DT*^*CCS*_*N2,ref*_ values were determined (see Table S4). The mixture of SIL steroids was added to all samples to yield a final concentration of 0.5 mg/L in each vial.

### Comparison of datasets

Bias between new experimental data and literature values were calculated as follows:

1 $$Bias \%=\frac{{{CCS}_{N2,exp}-CCS}_{N2,ref}}{{CCS}_{N2,ref}}$$

A summary of new experimental data recorded and datasets from literature used for comparison is provided in Table 1.

**Table 1:** Summary of new experimental and literature datasets used for assessment of different calibration approaches for TWIM-MS within the present work

Dataset	Reference	Information
SL*	Hernández-Mesa et al. [7, 15]	Single laboratory ^*TW*^*CCS*_*N2*_ values (Synapt G2 S)
IL*	Hernández-Mesa et al. [13]	Interlaboratory ^*TW*^*CCS*_*N2*_ values (average from four TWIM-MS instruments)
A	New experimental data	^*TW*^*CCS*_*N2*_ calibration with Agilent ESI-L tune mix
B, B2	New experimental data	^*TW*^*CCS*_*N2*_ calibration with new ^*DT*^*CCS*_*N2,ref*_ for Waters CCS Major Mix
ST	New experimental data	^*TW*^*CCS*_*N2*_ calibration with new ^*DT*^*CCS*_*N2,ref*_ for steroids as calibrants
ST-SIL, B2-SIL	New SIL-corrected data	^*TW*^*CCS*_*N2*_ values after correction using SIL-information (see Electronic Supplementary Information Table S13)
^*DT*^*CCS*_*N2*_ values**	Feuerstein et al. [14]	^*DT*^*CCS*_*N2*_ values from Agilent 6560 DTIM-MS
^*TIM*^*CCS*_*N2*_ values**	Feuerstein et al. [14]	^*TIM*^*CCS*_*N2*_ values from Bruker timsTOF pro

*Generated using the recommended *CCS* calibration strategy using Waters CCS Major Mix

**Generated using the recommended *CCS* calibration using Agilent ESI-L tune mix

### Computational methods

Gaussian 16 software was used for DFT calculations. Ion structures were fully optimized by density functional theory (DFT) with B3LYP and wB97xD functionals. The basis set 6–311 +  + G(d,p) including both diffuse and polarization functions was used for the calculations. Frequency calculations were performed at the same level of theory at 298.15 K to find optimized structures for local minima. Charge distribution was calculated using the Merz-Kollman (MK) method. The Gaussian output files containing geometrical parameters of the candidate structures and MK charges were used to build input files for *CCS*_*N2*_ calculations. *CCS*_*N2*_ calculations were performed using MOBCAL-MPI software using the trajectory method (TM) [33, 34]. *CCS*_*N2*_ values were predicted for 298 K in 10 cycles. Velocity integration was set to 48 and impact integration was set to 512 in the graphical user interface.

## Results and discussion

### Investigations of systematic error from current external calibration for IM-MS

In our previous research, we could demonstrate a systematic bias in single laboratory and interlaboratory ^*TW*^*CCS*_*N2*_ values compared to corresponding ^*DT*^*CCS*_*N2*_ or ^*TIM*^*CCS*_*N2*_ values. Because both DTIM-MS and TIM-MS instruments are calibrated with the same set of calibrant ions and reference values while the TWIM-MS systems are calibrated with different calibrants and reference *CCS*_*N2*_ values, the influence of the external calibration was hypothesized as a major contribution to this observed bias [14]. To further investigate this hypothesis, CCS Major Mix was analysed using an Agilent 6560 DTIM-MS, and new ^*DT*^*CCS*_*N2*_ values were compared to the reference values that are routinely applied for TWIM-MS (referred to as *CCS*_*N2,ref*_ in this work).

**Fig. 1:**
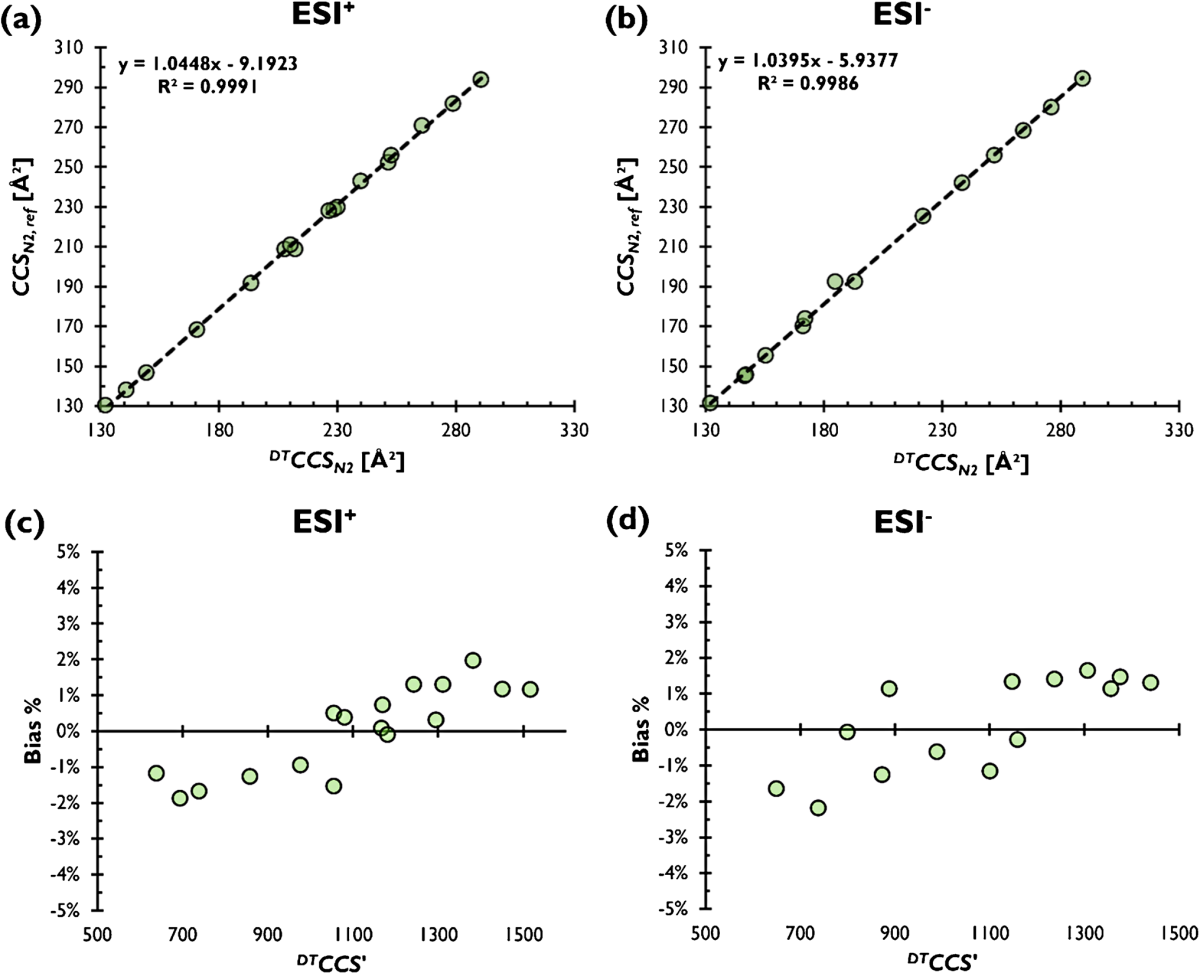
Linear models comparing standard *CCS*_*N2, ref*_ values of CCS Major Mix with ^*DT*^*CCS*_*N2*_ of the same ions determined experimentally with DTIM-MS **a** in ESI^+^ and **b** ESI^−^ mode. Bias between *CCS*_*N2,ref*_ and new experimental ^*DT*^*CCS*_*N2*_ values with respect to ^*DT*^*CCS’* for **c** ESI^+^ and **d** ESI^−^ modes

Systematic errors depending on *CCS’* were observed in both polarities (Fig. 1). Additionally, the intercepts and coefficients of the obtained linear models resemble the linear fits for the steroid data in our previous study in a direct comparison of ^*DT*^*CCS*_*N2*_ with the reference values (see Fig. 1, Table S1 and Table S2) [14]. It was also noteworthy that some of the investigated calibrant ions exhibited non-uniform arrival time distributions on the DTIM-MS system, which may influence their reliability of *CCS*_*N2*_ calibration particularly for high-resolution IM-MS (see Figure S1). Given that the resolution of most current IM-MS instrumentation does not permit full resolution of these apparent conformers, software-based peak picking results from DTIM-MS data with native resolution (50–60) were used for further work.

### Evaluation of alternative external calibration strategies for TWIM-MS

To delineate the influence of the external calibration bias arising from the selection of *CCS*_*N2,ref*_ values from other influencing factors (e.g. ion heating or instrument-specific effects such as source conditions), alternative approaches for external TWIM-MS calibration were experimentally assessed. Three strategies were tested using IM-MS database values from 87 steroids: (1) Agilent ESI-L tune mix ions for ^*TW*^*CCS*_*N2*_ calibration, (2) use of new reference values for Waters CCS Major Mix determined with the Agilent 6560 DTIM-MS for calibration and (3) use of a new set of class-specific calibrant ions and reference values established. Full details and tables of reference ^*DT*^*CCS*_*N2*_ values used for these calibration strategies are presented in Tables S1–S3.

**Fig. 2:**
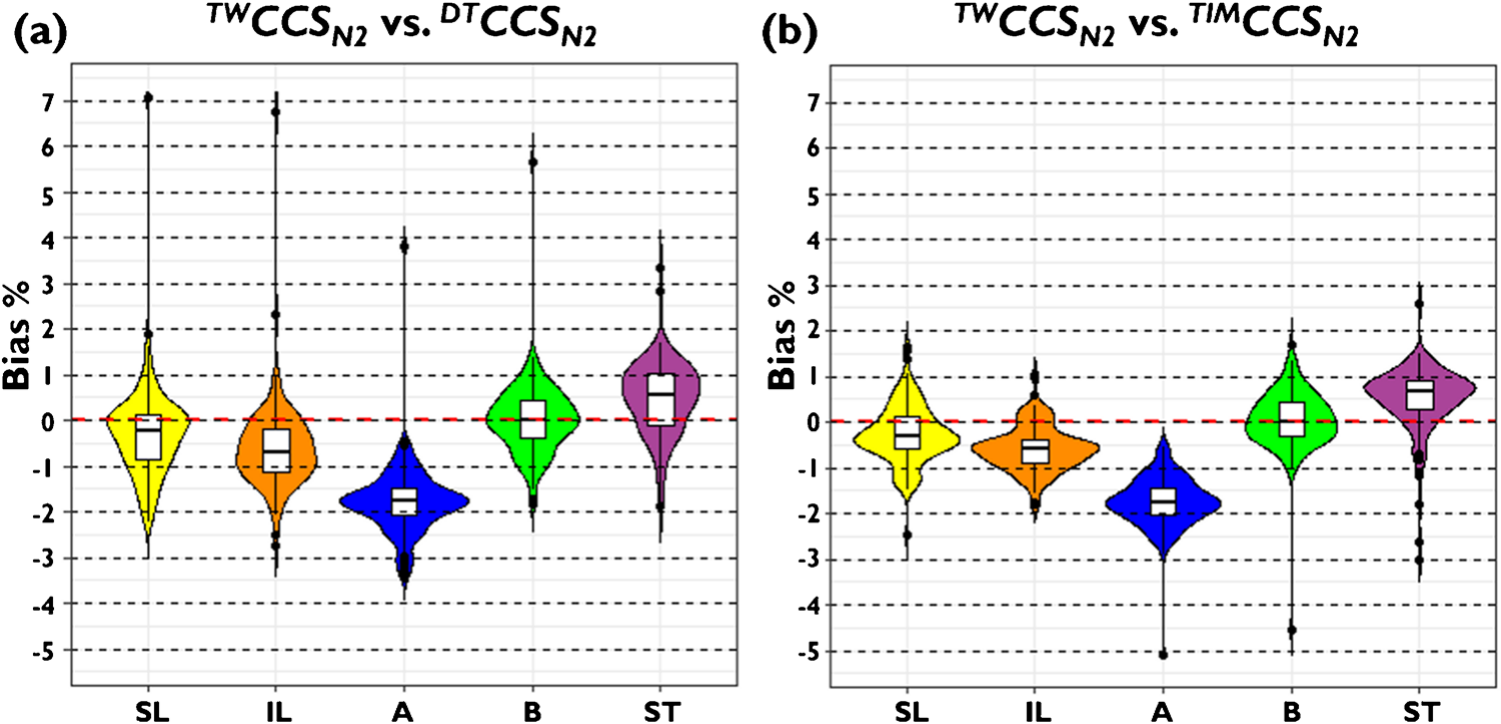
Bias data according to applied external calibration strategies compared to **a** ^*DT*^*CCS*_*N2*_ and **b** ^*TIM*^*CCS*_*N2*_ values with respect to published single laboratory data (SL) [15] and interlaboratory data (IL) [13] that employed the vendor-recommended procedure for ^*TW*^*CCS*_*N2*_ calibration. Shown alongside are new experimental TWIM-MS data calibrated using the Agilent ESI-L tune mix approach (A), newly determined ^*DT*^*CCS*_*N2*_ reference for CCS Major mix (B); and class-specific external calibrant ions (ST)

The bias of derived ^*TW*^*CCS*_*N2*_ values from each approach was compared to previously published ^*DT*^*CCS*_*N2*_ (Fig. 2a) and ^*TIM*^*CCS*_*N2*_ values as references (Fig. 2b and Table 2). The smallest average systematic bias compared to ^*DT*^*CCS*_*N2*_ (+ 0.02%) and ^*TIM*^*CCS*_*N2*_ values (+ 0.05%) was obtained by using Waters CCS Major Mix with newly established ^*DT*^*CCS*_*N2*_ reference values, whereby 95% of the values were within 1.29% of ^*DT*^*CCS*_*N2*_ values and within 1.12% of ^*TIM*^*CCS*_*N2*_ values. Furthermore, only a single ion ([M-H]^−^ ion of estradiol diglucuronide) remained with a bias of greater than 2% compared to the corresponding ^*DT*^*CCS*_*N2*_ value, while only boldenone undecylenate [M + Na]^+^ was outside of ± 2% range compared to the corresponding ^*TIM*^*CCS*_*N2*_ value. In contrast, the other investigated external calibration strategies were found to not perform better than the vendor-recommended calibration procedure that was applied in previous single (SL) and interlaboratory (IL) TWIM-MS studies. In particular, use of the Agilent ESI-L tune mix was found to be unsuitable for reliable TWIM-MS calibration resulting in large negative systematic average bias of approximately − 1.8% compared to both ^*DT*^*CCS*_*N2*_ and ^*TIM*^*CCS*_*N2*_ datasets, which is in agreement with a recent report on calibration of a novel TWIM-based high-resolution SLIM-MS device for the analysis of lipids [35]. Finally, using steroids as class-specific calibrant ions for TWIM-MS datasets resulted in a positive bias of approximately 0.5% compared to both ^*DT*^*CCS*_*N2*_ and ^*TIM*^*CCS*_*N2*_ datasets. While the class-specific approach performed much better than the unified calibrant approach with Agilent ESI-L tune mix, the low coverage of the calibration range and small number of datapoints appear as a major limitation for this approach for TWIM-MS calibration where the relationship between *CCS’* and arrival time is non-linear. Additionally, the number of datapoints in the relevant *CCS’* range with Agilent ESI-L tune mix is used is low (see Figure S2).

**Table 2:** Bias and absolute bias of ^*TW*^*CCS*_*N2*_ compared to ^*DT*^*CCS*_*N2*_ (*n* = 132–134) and ^*TIM*^*CCS*_*N2*_ (*n* = 134–139) values for different external calibration approaches studied in this work

Dataset	Bias % (vs. ^*DT*^*CCS*_*N2*_)		Abs. Bias % (vs. ^*DT*^*CCS*_*N2*_)		Bias % (vs. ^*TIM*^*CCS*_*N2*_)		Abs. Bias % (vs. ^*TIM*^*CCS*_*N2*_)	
	Average	SD	Average	95th perc	Average	SD	Average	95th perc
SL	− 0.30%	1.02%	0.70%	1.87%	− 0.27%	0.62%	0.54%	1.34%
IL	− 0.58%	0.94%	0.79%	1.91%	− 0.58%	0.48%	0.64%	1.34%
A	− 1.78%	0.77%	1.83%	3.04%	− 1.75%	0.55%	1.75%	2.54%
B	0.02%	0.80%	0.54%	1.29%	0.05%	0.66%	0.45%	1.12%
ST	0.48%	0.79%	0.76%	1.56%	0.50%	0.73%	0.74%	1.43%

**Fig. 3:**
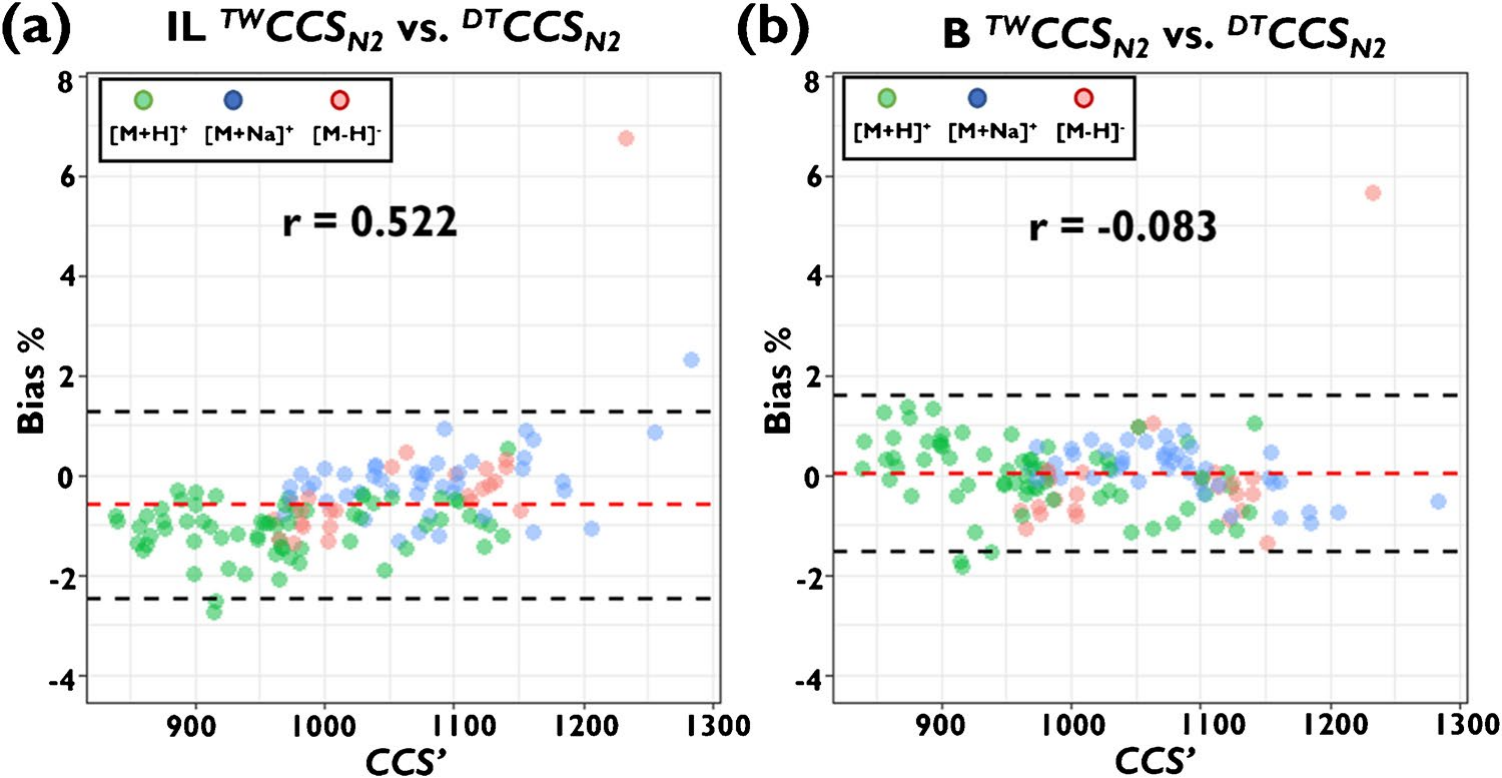
Bland–Altman diagrams showing bias between ^*DT*^*CCS*_*N2*_ (= ref.) and ^*TW*^*CCS*_*N2*_ values determined as **a** interlaboratory averages [13] and **b** using newly determined ^*DT*^*CCS*_*N2*_ reference values for CCS Major Mix calibrant ions. Bias data are shown according to the separation order (*CCS’*). Dashed lines shown indicate average bias (red) and ± 1.96 the standard deviation (black), respectively. *r* is the Pearson correlation coefficient

The effect of applying a new calibration on the observed *CCS’* dependency of reported bias in a large dataset was further investigated using Pearson correlation (Fig. 3). While a moderate positive correlation of the bias between ^*DT*^*CCS*_*N2*_ values and the interlaboratory dataset with respect to *CCS’* is apparent (Pearson *r* = 0.535), this correlation could be diminished after calibration with new DTIM-MS reference values for CCS Major Mix (Pearson *r* =  − 0.083). This improvement was found to benefit specific examples in the dataset including the [M + Na]^+^ ion of boldenone undecylenate, which exhibited improved agreement with recently established ^*DT*^*CCS*_*N2*_ [14] using the calibration approach with new reference values for CCS Major Mix (see Table S12). Previously, a bias of 2.3% between interlaboratory ^*TW*^*CCS*_*N2*_ and ^*DT*^*CCS*_*N2*_ was observed, but this was reduced to 0.5% using the new reference values for TWIM-MS calibration indicating that the same ion conformation appears to be sampled on both DTIM-MS and TWIM-MS.

### Evaluation of internal correction using stable isotope labelled standards

As the establishment of new reference values for IM-MS calibrants for routine *CCS* determination is not a trivial task, further analytical strategies to reduce bias between datasets were considered. One such candidate method is the employment of correction functions based on a set of internal standards spiked into all samples to be used for multiple correction functions or for internal calibrations [35, 36]. While the use of natural internal standards limits the number of compounds that can be spiked, SIL-based internal standardization are ideal candidates for application to LC-IM-MS methods by exploiting the alignment of isotopologues in both LC and IM dimensions [18, 37, 38]. Due to their frequent use as internal standards for quantitative purposes, the potential of increasing the scope of this approach to include an internal correction for *CCS*_*N2*_ determination was also considered here for the first time for steroid analysis. To this end, ^*DT*^*CCS*_*N2*_ values were established for SIL-compounds and used to monitor bias of externally calibrated ^*TW*^*CCS*_*N2*_ values (Table S4). The ratios of measured ^*TW*^*CCS*_*N2*_ values and ^*DT*^*CCS*_*N2*_ were then used to monitor systematic bias trends as a function of *CCS’* and allow derivation of correction factors from linear models to be used as empirical correction factors applied to experimental ^*TW*^*CCS*_*N2*_ values (Figure S3); and results are presented in detail in Figure S4 and Table S13**.** One major observation is that due to the broad bias distributions encountered in all externally calibrated datasets (i.e. standard deviations between 0.5% and 1.1% were observed), a sufficiently large number of SIL internal standards appears to be necessary for internal correction strategies to achieve appropriate correction of calibration-dependent systematic bias. This is a practical challenge for many applications as SIL internal standards are typically expensive, and availability is limited or non-existent for some molecular classes. This was found to be true in the case of negative mode for this application where only two suitable SIL-steroid compounds (one sterol-sulphate and one sterol-glucuronide) could be employed in this study. Therefore, SIL-based internal correction was only applied to protonated and sodiated ions in ESI^+^ data (Table 3).

**Table 3:** Bias and absolute bias of ^*TW*^*CCS*_*N2*_ compared to ^*DT*^*CCS*_*N2*_ (*n* = 107) and ^*TIM*^*CCS*_*N2*_ (*n* = 109) for class-specific external calibration (ST) and newly determined ^*DT*^*CCS*_*N2*_ reference values for CCS Major Mix (B2) followed by corresponding internal correction of ^*TW*^*CCS*_*N2*_ values using SIL steroids and linear models for determination of correction factors (ST-SIL and B2-SIL, respectively)

Dataset	Bias % (^*DT*^*CCS*_*N2*_ = ref.)		Abs. Bias % (^*DT*^*CCS*_*N2*_ = ref.)		Bias % (^*TIM*^*CCS*_*N2*_ = ref.)		Abs. Bias % (^*TIM*^*CCS*_*N2*_ = ref.)	
	Average	SD	Average	95th perc	Average	SD	Average	95th perc
ST	0.61%	0.69%	0.79%	1.54%	0.63%	0.63%	0.75%	1.44%
ST-SIL	0.12%	0.73%	0.54%	1.37%	0.13%	0.72%	0.54%	1.26%
B2	-0.08%	0.59%	0.45%	1.19%	-0.06%	0.61%	0.41%	0.87%
B2-SIL	-0.06%	0.69%	0.52%	1.29%	-0.04%	0.58%	0.41%	0.95%

This correction strategy was applied to datasets that were externally calibrated using the native steroid mix (ST-SIL) and using newly determined ^*DT*^*CCS*_*N2*_ values as reference for the routinely used CCS Major Mix (B2-SIL). Prior to application of the correction, a systematic positive bias for the ST dataset was observed (0.61% ± 0.69% compared to ^*DT*^*CCS*_*N2*_ data), while the systematic bias was negligible for the B2 dataset (− 0.08% ± 0.59% compared to ^*DT*^*CCS*_*N2*_ data). Application of SIL-based correction was found to reduce the average absolute bias of ST dataset with respect to both corresponding ^*DT*^*CCS*_*N2*_ and ^*TIM*^*CCS*_*N2*_ data (Figure S4). The significance of this improvement was tested using a non-parametric Wilcoxon test, which revealed that the improvement from the SIL-based correction of the ST data (ST-SIL) was significant (*p* < 0.05), whereas the corresponding change of the bias distribution in B2 data (B2-SIL) was not significant (*p* > 0.05, see Figure S4). The effect of internal correction was found to be negligible and good agreement with ^*DT*^*CCS*_*N2*_ and ^*TIM*^*CCS*_*N2*_ with 95th percentiles in the range of 1.0–1.3% was maintained (see Table 3). The difference between the B2-SIL data and ST-SIL data was also investigated and was found to be significant (see Figure S5). Taken together, these results demonstrate that, while the internal correction method based on SIL analogues can reduce systematic bias in such datasets and has potential for method-specific application across different IM-MS platforms, standardization of external calibration strategies remains the most critical issue for *CCS* determination using TWIM-MS. Rose et al. [35] noted similar observations during optimization of the calibration procedure of a high-resolution SLIM-MS device for the analysis of lipids. The results also highlight challenges faced in calibration of TWIM-based technology and the need for optimization of external calibration approaches for the calibration of new IM-MS technologies used for small molecule analysis.

### Understanding outliers using in silico calculations

While the application of alternative calibration and internal correction approaches was shown in this work to minimize average and systematic bias between ^*TW*^*CCS*_*N2*_ data and reference *CCS*_*N2*_ data from both DTIM-MS and TIM-MS instruments, a small subset of outliers within the individual datasets remained. These now almost unambiguous outlier values may represent true conformational differences of the corresponding ions sampled on the different instruments or may be result of more complex behaviour such as dissociation or intermediate complex formation. To investigate these outliers in detail, DFT calculations were used to determine the structures of possible conformers, protomers, and deprotomers of some of these outliers, followed by *CCS*_*N2*_ prediction for the candidate geometries using MOBCAL-MPI software [34].

The two methods used for structural optimization (B3LYP and ωB97xD functionals) can yield differences in optimized geometries, particularly for ions with flexible structures and considering the inclusion of atom–atom dispersion corrections in ωB97xD [39]. Thus, to benchmark performance of the employed workflow, *CCS*_*N2*_ values of several common reference ions used routinely for IM-MS calibration were calculated for the structures optimized by these two functionals. As each ion can have several conformers or (de)protomers, structural optimization, charge distribution analysis, and *CCS*_*N2*_ calculations were carried out for all these isomers. The ωB97xD-optimized structures of several common tune ions and the relative stabilities of conformers and (de)protomers were compared using the calculated Gibbs free energies (Figures S6–S10) and predicted *CCS*_*N2*_ values for the most stable candidate geometries are compared with the experimentally determined ^*DT*^*CCS*_*N2*_ in Tables S5–S9. Overall, the ωB97xD-predicted *CCS*_*N2*_ were found to be in better agreement with the experimental values, but uncertainty with such predictions remains large. All candidate geometries and corresponding *CCS*_*N2*_ values for the [M + H]^+^ ions of acetaminophen and verapamil could nevertheless be tentatively correlated to the experimental DTIM-MS spectra presenting non-uniform distributions for these ions (Figure S1).

**Fig. 4:**
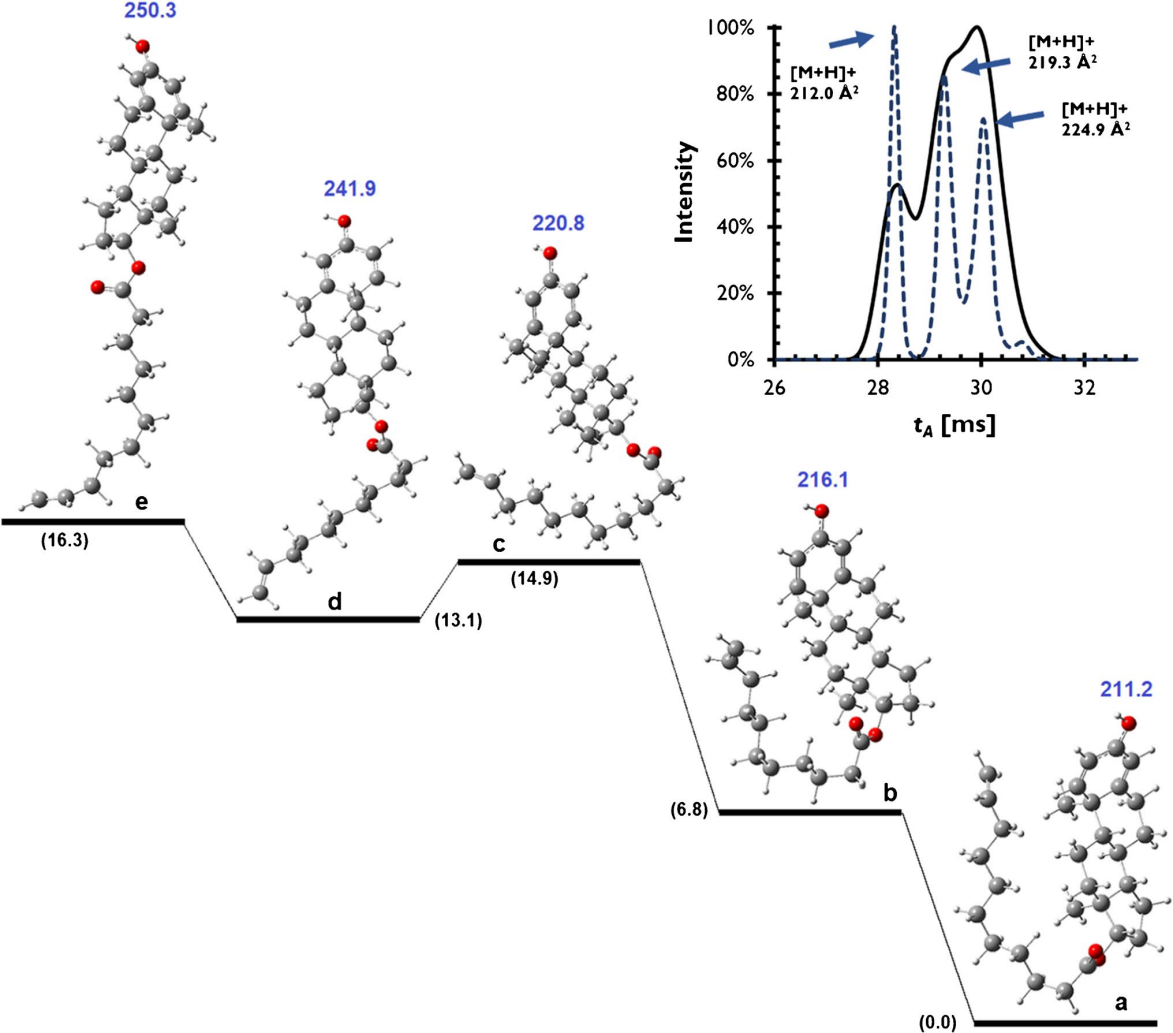
The Gibbs free energy diagram and optimized structures for five of the most stable conformers of [BU + H]^+^. The relative Gibbs energies (numbers in parenthesis) and calculated *CCS* values are in kJ mol^−1^ and Å^2^, respectively. The insert shows arrival time spectra determined using DTIM-MS using 4-bit multiplexing (solid line) and high-resolution demultiplexing (dashed line) [14]

Both the protonated and sodiated adducts of boldenone undecylenate (BU) showed unexpected IM behaviours in DT, TW, and TIM. As only the protonated ion was experimentally observed with good abundance with all instrument platforms, this was the focus for additional computational predictions. The optimized candidate geometries and relative energies of [BU + H]^+^ are shown in Fig. 4. The small difference between the Gibbs free energies of the candidate geometries indicates multiple possible candidate structures for this ion. In the absence of any external collision or energy, the Boltzmann distribution at 298 K for the conformers **a**, **b**, **c**, **d** and **e** is as 93.2%, 6.0%, 0.2%, 0.5% and 0.1%, respectively. While these predictions allow rationalization of experimental results, the observed distribution of the conformers is expected to depend on the ion source geometry and conditions (i.e. temperatures, voltages) experienced by the ion on the respective IM-MS platforms even when all other analytical method parameters (i.e. LC flow rate, solvent composition) are kept consistent. This is a major challenge for development of interplatform *CCS* databases covering compounds with a high degree of flexibility leading to complex arrival time distributions where multiple *CCS* values cannot routinely be compared due to both the differences in ion source conditions and resolving power of different IM-MS platforms.

In the negative mode, the measured *CCS*_*N2*_ values for estradiol diglucuronide, [ED-H]^−^, in DTIM-MS, TWIM-MS and TIM-MS are 238.2, 254.3 and 253.8 Å^2^, respectively [14]. Due to the structure of this compound with its two glucuronic acid groups, the occurrence of two distinct deprotomers with different *CCS* is a possible explanation for the differences observed between the different instruments. DFT calculations showed that while both deprotomers (see [ED-H]^−^-a and [ED-H]^−^-b in Figure S11) can be formed in ESI^−^, the predicted *CCS*_*N2*_ for these deprotomers differed by only 1.3% (see Table S10) indicating that differences observed between the experimental *CCS*_*N2*_ from different IM-MS platforms for [ED-H]^−^ are too large to be interpreted as distinct deprotomers being observed on different types of IM-MS instruments. Therefore, further conformations of [ED-H]^−^ were also considered. The calculated *CCS*_*N2*_ values for the conformers of this ion (Table S10, conformers c-h) are in the range of 241.0 Å^2^ (most compact) up to 283.8 Å^2^ (most open) which makes unambiguous correlation with experimental *CCS*_*N2*_ values from the different IM-MS platforms challenging. Thus, to further benchmark the significance of these results, we also calculated the theoretical *CCS*_*N*2_ for another experimentally observed anion of ED, i.e. [ED-2H + Na]^−^ for which the measured *CCS*_*N2*_ in DTIM-MS, TWIM-MS and TIM-MS were very similar (239.6, 240.1 and 238.3 Å^2^, respectively). The optimized structures of different conformers of [ED-2H + Na]^+^ are shown in Figure S12. Similar to [ED-H]^−^, a large difference between the theoretical (Table S11) and experimental *CCS*_*N2*_ of [ED-2H + Na]^−^ was found for the open conformations, which suggests that a compact conformation of this ion is experimentally observed, e.g. a *CCS*_*N2*_ of 252 Å^2^ was predicted for conformer f. The greater stability due to lower Gibbs energy (~ 70–100 kJ/mol) of the closed conformation is thus a plausible explanation for the stability of this conformer across different IM-MS platforms. Therefore, one plausible explanation for the [ED-H]- results is that, despite being energetically less favourable, the relatively small energetic difference (~ 15 kJ/mol) may allow formation of the closed conformer for [ED-H]^−^ with a *CCS*_*N2*_ of 241.0 Å^2^ in the ESI source of the used DTIM-MS instrument platform, rather than one of the open conformations.

Finally, as exhaustive review of the DTIM-MS data acquired with different measurement conditions could not provide conformation of this first hypothesis, a second feasible origin of the discrepancies between IM-MS platforms is ion transport effects occurring on the TWIM-MS and TIM-MS platforms which were also considered. A plausible mechanism would involve formation and transport of multimeric species that are dissociated in a post-IM region and then detected as [ED-H]^−^. Some experimental evidence supporting this hypothesis from DTIM-MS data is presented in Figure S13. Overall, these results highlight the difficulty of unambiguously correlating *CCS* predictions with experimental values due not only to previously reported issues with predictions using nitrogen as drift gas [40–42], but also the fundamental challenge of correctly optimizing candidate geometries and the additional potential for non-ideal behaviour such as clustering that can lead to major discrepancies between *CCS* values reported on different IM-MS platforms.

## Conclusion

Investigation of calibration-dependent bias and testing of alternative calibration sets for TWIM-MS in this work highlights the critical importance of external calibration for *CCS*_*N2*_ determination using IM-MS. While good agreement between different types of IM-MS was shown in previous research for steroid analysis (i.e. bias < 2% for most investigated compounds), use of new *CCS*_*N2,ref*_ values for routinely used TWIM-MS calibrant ions is shown to be best-suited for amelioration of the *CCS*’-dependent trends observed with respect to both DTIM-MS and TIM-MS. This improvement is also of fundamental importance for differentiating ions with true structural differences observed on different instruments from outliers that are in fact resultant from calibrant-dependent effects.

Other analytical strategies investigated in this work showed limitations for the application investigated. A unified calibration with Agilent ESI-L tune mix and a class-specific calibration mixture (steroids) could not improve the average bias between ^*TW*^*CCS*_*N2*_ and reference *CCS*_*N2*_ values. However, a new approach using stable isotope labelled (SIL) internal standards to internally correct ^*TW*^*CCS*_*N2*_ data using ratios of ^*DT*^*CCS*_*N2*_ values and measured ^*TW*^*CCS*_*N2*_ values of internal standards significantly improved agreement between datasets from different IM-MS platforms. Although this SIL-based approach can be cost-prohibitive and cannot replace proper external ^*TW*^*CCS*_*N2*_ calibration, it may be a candidate method that can be applied across IM-MS platforms for specific applications.

DFT calculations in combination with *CCS*_*N2*_ prediction could provide rational explanations for some experimental observations according to alternative ion conformations, flexibility of side chains or the formation of multimeric ion clusters for some ions. Although a detailed mechanistic understanding of observed differences is not always possible due to the relatively high uncertainties associated with such in silico predicted *CCS* values, such methods are valuable to test hypotheses for individual examples in small molecule IM-MS datasets.

## Supplementary Information

Below is the link to the electronic supplementary material.

Supplementary file1 (DOCX 3536 kb)

Supplementary file2 (XLSX 106 kb)

## References

[1] Schoeny H, Rampler E, Binh Chu D, Schoeberl A, Galvez L, Blaukopf M (2022). Achieving absolute molar lipid concentrations: a phospholipidomics cross-validation study. Anal Chem.

[2] Zhang X, Kew K, Reisdorph R, Sartain M, Powell R, Armstrong M (2017). Performance of a high-pressure liquid chromatography-ion mobility-mass spectrometry system for metabolic profiling. Anal Chem.

[3] Ortmayr K, Causon TJ, Hann S, Koellensperger G (2016). Increasing selectivity and coverage in LC-MS based metabolome analysis. TrAC Trends Anal Chem.

[4] Mairinger T, Causon TJ, Hann S (2018). The potential of ion mobility–mass spectrometry for non-targeted metabolomics. OMICS.

[5] Venter P, Muller M, Vestner J, Stander MA, Tredoux AGJ, Pasch H (2018). Comprehensive three-dimensional LC × LC × ion mobility spectrometry separation combined with high-resolution ms for the analysis of complex samples. Anal Chem.

[6] Causon TJ, Hann S (2015). Theoretical evaluation of peak capacity improvements by use of liquid chromatography combined with drift tube ion mobility-mass spectrometry. J Chromatogr A..

[7] Hernández-Mesa M, Monteau F, Le Bizec B, Dervilly-Pinel G (2019). Potential of ion mobility-mass spectrometry for both targeted and non-targeted analysis of phase II steroid metabolites in urine. Anal Chim Acta X.

[8] Feuerstein ML, Hann S, Causon T. Chapter 7 ion mobility–time-of-flight mass spectrometry and applications for metabolomics. In: Advanced Mass Spectrometry-based Analytical Separation Techniques for Probing the Polar Metabolome. R Soc of Chem. 2021;165–84. 10.1039/9781839163524-00165.

[9] May JC, McLean JA (2015). Ion mobility-mass spectrometry: time-dispersive instrumentation. Anal Chem..

[10] Dodds JN, Baker ES (2019). Ion mobility spectrometry: fundamental concepts, Instrumentation, Applications, and the Road Ahead. J Am Soc Mass Spectrom.

[11] Gabelica V, Shvartsburg AA, Afonso C, Barran P, Benesch JLP, Bleiholder C (2019). Recommendations for reporting ion mobility mass spectrometry measurements. Mass Spectrom Rev..

[12] Stow SM, Causon TJ, Zheng X, Kurulugama RT, Mairinger T, May JC (2017). An Interlaboratory evaluation of drift tube ion mobility-mass spectrometry collision cross section measurements. Anal Chem..

[13] Hernández-Mesa M, D’Atri V, Barknowitz G, Fanuel M, Pezzatti J, Dreolin N (2020). Interlaboratory and interplatform study of steroids collision cross section by traveling wave ion mobility spectrometry. Anal Chem.

[14] Feuerstein ML, Hernández-Mesa M, Kiehne A, Le Bizec B, Hann S, Dervilly-Pinel G, et al. Comparability of steroid collision cross sections using three different IM-HRMS technologies: an interplatform study. ChemRxiv. 2022; 10.26434/chemrxiv-2022-87k68 10.1021/jasms.2c00196 PMC9545150 36047677

[15] Hernández-Mesa M, Le Bizec B, Monteau F, García-Campaña AM, Dervilly-Pinel G (2018). Collision cross section (CCS) database: an additional measure to characterize steroids. Anal Chem.

[16] Picache JA, Rose BS, Balinski A, Leaptrot KL, Sherrod SD, May JC (2018). Collision cross section compendium to annotate and predict multi-omic compound identities. Chem Sci.

[17] Belova L, Caballero-Casero N, van Nuijs ALN, Covaci A (2021). Ion mobility-high-resolution mass spectrometry (IM-HRMS) for the analysis of contaminants of emerging concern (CECS): database compilation and application to urine samples. Anal Chem.

[18] Feuerstein ML, Kurulugama RT, Hann S, Causon T (2021). Novel acquisition strategies for metabolomics using drift tube ion mobility-quadrupole resolved all ions time-of-flight mass spectrometry (IM-QRAI-TOFMS). Anal Chim Acta.

[19] da Silva KM, Iturrospe E, Heyrman J, Koelmel JP, Cuykx M, Vanhaecke T (2021). Optimization of a liquid chromatography-ion mobility-high resolution mass spectrometry platform for untargeted lipidomics and application to HepaRG cell extracts. Talanta.

[20] Causon TJ, Hann S. Uncertainty estimations for collision cross section determination via uniform field drift tube-ion mobility-mass spectrometry. J Am Soc Mass Spectrom. 2020. 10.1021/jasms.0c00233. 10.1021/jasms.0c00233 32812758

[21] Hines KM, May JC, McLean JA, Xu L (2016). Evaluation of collision cross section calibrants for structural analysis of lipids by traveling wave ion mobility-mass spectrometry. Anal Chem.

[22] Kwantwi-Barima P, Harrilal CP, Garimella SVB, Attah IK, Smith RD, Ibrahim YM (2022). Effect of traveling waveform profiles on collision cross section measurements in structures for lossless ion manipulations. J Am Soc Mass Spectrom.

[23] Davis DE, Leaptrot KL, Koomen DC, May JC, Cavalcanti G de A, Padilha MC, et al. Multidimensional separations of intact phase II steroid metabolites utilizing LC–ion mobility–HRMS. Anal Chem. 2021;93(31):10990–8. 10.1021/acs.analchem.1c02163 PMC9288154 34319704

[24] Velosa DC, Rivera ME, Neal SP, Olsen SSH, Burkus-Matesevac A, Chouinard CD (2022). Toward routine analysis of anabolic androgenic steroids in urine using ion mobility-mass spectrometry. J Am Soc Mass Spectrom.

[25] Hinnenkamp V, Klein J, Meckelmann SW, Balsaa P, Schmidt TC, Schmitz OJ (2018). Comparison of CCS values determined by traveling wave ion mobility mass spectrometry and drift tube ion mobility mass spectrometry. Anal Chem..

[26] Graton J, Hernández-Mesa M, Normand S, Dervilly G, Le Questel JY, Le Bizec B (2020). Characterization of steroids through collision cross sections: contribution of quantum chemistry calculations. Anal Chem.

[27] Bilbao A, Gibbons BC, Stow SM, Kyle JE, Bloodsworth KJ, Payne SH, et al. A Preprocessing tool for enhanced ion mobility–mass spectrometry-based omics workflows. J Proteome Res. 2021. 10.1021/acs.jproteome.1c00425. 10.1021/acs.jproteome.1c00425 PMC8837709 34382401

[28] Tsugawa H, Cajka T, Kind T, Ma Y, Higgins B, Ikeda K (2015). MS-DIAL: data-independent MS/MS deconvolution for comprehensive metabolome analysis. Nat Methods.

[29] Tsugawa H, Ikeda K, Takahashi M, Satoh A, Mori Y, Uchino H, et al. A lipidome atlas in MS-DIAL 4. Nat Biotechnol. 2020. 10.1038/s41587-020-0531-2. 10.1038/s41587-020-0531-2 32541957

[30] Ruotolo BT, Benesch JLP, Sandercock AM, Hyung SJ, Robinson CV (2008). Ion mobility–mass spectrometry analysis of large protein complexes. Nat Protoc.

[31] R Core Team. R: A language and environment for statistical computing. Vienna, Austria: R Foundation for Statistical Computing. 2021. Available from: https://www.R-project.org/. Accessed 01 Dec 2021.

[32] RStudio Team. RStudio: integrated development environment for R. Boston, MA: RStudio, PBC. 2020. Available from: http://www.rstudio.com/. Accessed 01 Dec 2021.

[33] Ieritano C, Hopkins WS (2021). Assessing collision cross section calculations using MobCal-MPI with a variety of commonly used computational methods. Mater Today Commun.

[34] Ieritano C, Crouse J, Campbell JL, Hopkins WS (2019). A parallelized molecular collision cross section package with optimized accuracy and efficiency. Analyst.

[35] Rose BS, May JC, Reardon AR, McLean JA. Collision cross-section calibration strategy for lipid measurements in SLIM-based high-resolution ion mobility. J Am Soc Mass Spectrom. 2022. 10.1021/jasms.2c00067 10.1021/jasms.2c00067 PMC9516683 35653638

[36] Manz C, Götze M, Frank C, Zappe A, Pagel K. Dextran as internal calibrant for N-glycan analysis by liquid chromatography coupled to ion mobility-mass spectrometry. Anal Bioanal Chem. 2022. 10.1007/s00216-022-04133-0 10.1007/s00216-022-04133-0 PMC9234027 35614231

[37] Domenick TM, Jones AL, Kemperman RHJ, Yost RA. A rapid and robust method for amino acid quantification using a simple N-hydroxysuccinimide ester derivatization and liquid chromatography-ion mobility-mass spectrometry. Anal Bioanal Chem. 2022. 10.1007/s00216-022-03993-w 10.1007/s00216-022-03993-w 35338375

[38] Dodds JN, Wang L, Patti GJ, Baker ES (2022). Combining isotopologue workflows and simultaneous multidimensional separations to detect, identify, and validate metabolites in untargeted analyses. Anal Chem.

[39] Chai JD, Head-Gordon M (2008). Long-range corrected hybrid density functionals with damped atom–atom dispersion corrections. Phys Chem Chem Phys.

[40] Chatterjee P, Dutta SS, Chakraborty T (2022). Tautomers and Rotamers of Curcumin: A combined UV spectroscopy, high-performance liquid chromatography, ion mobility mass spectrometry, and electronic structure theory study. J Phys Chem A.

[41] Bull JN, Scholz MS, Coughlan NJA, Bieske EJ (2017). Isomerisation of an intramolecular hydrogen-bonded photoswitch: protonated azobis(2-imidazole). Phys Chem Chem Phys.

[42] Bull JN, Coughlan NJA, Bieske EJ (2017). Protomer-specific photochemistry investigated using ion mobility mass spectrometry. J Phys Chem A.

