# Supplementary material for: Critical evaluation of the role of external calibration strategies for IM-MS
Source: Anal Bioanal Chem. 2022 Aug 12;414(25):7483–93. doi: 10.1007/s00216-022-04263-5 (PMC9482903; doi:10.1007/s00216-022-04263-5)
Supplement: Supplementary file 1 — Supplementary file1 (DOCX 3536 kb) [file 216_2022_4263_MOESM1_ESM.docx]

Supplementary information for: Critical evaluation of the role of external calibration strategies for IM-MS

Max L. Feuerstein^1^, Maykel Hernández-Mesa^2^, Younes Valadbeigi^1^, Bruno Le Bizec^2^, Stephan Hann^1^, Gaud Dervilly^2^, Tim Causon^1*^

---------------------------------------------------------------------------------------------------------

^1^ University of Natural Resources and Life Sciences, Vienna, Department of Chemistry, Institute of Analytical Chemistry, Muthgasse 18, 1190 Vienna, Austria

^2^ LABERCA, Oniris, INRAE, 44307 Nantes, France

*Corresponding author

Tel: +43 1 47654-77187

E-Mail: Tim.Causon@boku.ac.at

University of Natural Resources and Life Sciences, Vienna

Department of Chemistry

Institute of Analytical Chemistry

Muthgasse 18, Vienna, 1190

Austria

## DTIM-MS measurements

DTIM-MS measurements were performed on an Agilent 6560 IM-QTOFMS (Agilent Technologies, Santa Clara, CA) with a Dual AJS ESI Ion Source using direct infusion analysis or after separation with an Agilent 1290 Infinity II UHPLC system. For direct infusion measurements of standards, a syringe pump was used with flow rates of 1.8 mL/h and 1.2 mL/h using the single-field method. These LC conditions have been reported elsewhere(1,2). The source gas temperature was 225 °C, the gas flow was 8 L/min, the nebulizer pressure was 30 psi, sheath gas temperature and flow were 350°C and 12 L/min. The capillary voltage was 3500 V, and the nozzle voltage was 500 V. The mass range was set to 50-1700 m/z and measurements were performed using the 2 GHz Extended Dynamic Range setting. The drift gas pressure was 3.95 Torr with a temperature of 299.5 K. A drift tube entrance voltage of 1574 V and a drift tube exit voltage of 224 V were used. 4-bit multiplexing (4m) was used for data acquisition and 1.2 frames per second were recorded with 16 IM transients per frame. Trap fill time was set to 1250 µs and trap release time was 150 µs. External calibration files were recorded by infusing tune mix (Agilent ESI-L G1969-85000) for 30 s using the same acquisition settings as for samples. All calibration files underwent the same data pre-treatment (i.e., demultiplexing and smoothing) as the sample datafiles and were subsequently used for external calibration of datafiles using IM-MS Browser 10.0.

## DTIM-MS data analysis

4-bit multiplexed (LC-)DTIM-MS data was preprocessed with PNNL Preprocessor 3.0 (2021.04.21) and using the following workflow with for demultiplexing (“Step 2 (a): Multiplexed Data: Demux, Smooth, Spike Rem.”) and saturation repair (“Step 3: Saturation Repair”). Data was smoothed (moving average filters with a step-size set to 3), “Signal Intensity Lower Threshold” was set to 20 counts, spikes were removed and at least one adjacent data point per dimension was required. Furthermore, saturated peaks were repaired above an abundance level of 40%.

All DTIM-MS files were *CCS* calibrated using calibration coefficients determined in IM-MS Browser 10.0 from suitable calibration files. For peak picking using Mass Profiler 10.0 the charge state was limited to 1-2, retention time tolerances were ± 0.3 min, drift time tolerance was ±1%, mass tolerance was ± (15.0 ppm + 2.0 mDa) and a Q-score ≤ 70.0 was used to filter low-quality signals.

## TWIM-MS measurements

TWIM-MS measurements were performed in ESI^+^ and ESI^-^ mode using the following settings: full scan data was acquired in the mass range of 50-1200 *m*/*z* with a scan time of 0.2 s and data was acquired in high resolution mode. To allow use of Agilent ESI-L tune mix, data was acquired in a mass range of 50-1700 *m*/*z* for datasets calibrated with this calibrant mix (for both positive and negative modes). The capillary voltage was 2.5 kV in negative mode and 3.0 kV in positive mode, reference cone voltage was 40.0 V, sampling cone voltage was 31.0 V, source temperature was 150 °C, desolvation gas temperature was 350 °C, cone gas flow was 50 L/h, desolvation gas flow was 1000 L/h, nebulizer pressure was 6 bar. IMS bias was set to 3.0 V, trap collision energy was 4.0 V, transfer collision energy was 2.0 V, the IMS gas flow was 100 mL/min, helium gas flow was 180.0 mL/min, and the trap gas flow was 2.0 mL/min. The trap DC bias voltage was 45.0 V, wave velocity of step wave 1 was 300 m/s and wave height was 10.0 V, while wave height was 0.0 V for step wave 2, trap wave velocity was 311 m/s, and trap wave height was 4.0 V. IMS wave height was 40.0 V and the wave velocity was adapted to the employed calibrant ions; in ESI^+^ mode, wave velocity was set to 1000 m/s and in ESI^-^ wave velocity was set to 550 m/s, except for measurements using the Agilent ESI-L tune mix for *^TW^CCS_N2_* calibration whereby the wave velocity was set to 800 m/s. The transfer wave velocity was 219 m/s with a wave height of 4.0 V. The mobility trapping release time was 500 µs and mobility trap height was 15.0 V. Lock spray correction was performed using a 22 µL/min infusion of leucine enkephalin (1 ng/mL in 50:50 acetonitrile:water).

## MS-DIAL settings for TWIM-MS data

Prior to import into MS-DIAL, file conversion was performed using the MS-DIAL IBF-converter. Settings for the non-targeted peak picking and feature alignment in MS-DIAL were as follows. Mass tolerance of 0.01 Da was used for data extraction and alignment. Singly and doubly charged features in the mass range of 50-1200 *m/z* were accepted and a minimum peak height of 1000 counts was used. Data was smoothed using a linear moving average filter with box size of 3 and minimum peak width of 5 datapoints. Protonated, deprotonated, and sodiated adducts were accepted as potential ion species and data was aligned with a retention time tolerance of 0.2 min, a mass tolerance of 0.015 Da and a mobility tolerance of 0.02 ms. Arrival times were exported and *^TW^CCS_N2_* values were calculated in Microsoft Excel using corrected arrival times *t_c_* and calibration functions obtained using obtained from DriftScope (3).

## TWIM-MS calibration

In TWIM-calibration, a correction for charge state and reduced mass is used to yield a modified *CCS* (*CCS’*, Eq. 6).(4–6)

$CCS'= \frac{CCS\sqrt{\mu}}{z}$ (6)

Arrival times are then corrected for non-IM-related contributions to measured arrival times using an instrument dependent correction coefficient *C* (or enhanced duty cycle coefficient EDC) and the ions’ *m/z.*(4,5)

${t'}_{d}= t_{A}- \frac{C\sqrt{m/z}}{1000}$ (7)

Calibration coefficients *A* and *B* are then determined by fitting of logarithmic plot (Eq. 8) and *^TW^CCS_N2_* of unknown ions is calculated using a power function (Eq. 9).(4,5)

$\ln CCS'=B\cdot\ln{t^{'}}_{d}+\ln A$ (8)

$CCS=A\cdot{{t'}_{d}}^{B}\cdot\frac{z}{\sqrt{\mu}}$ (9)

## IM-MS Calibrant Ion Results


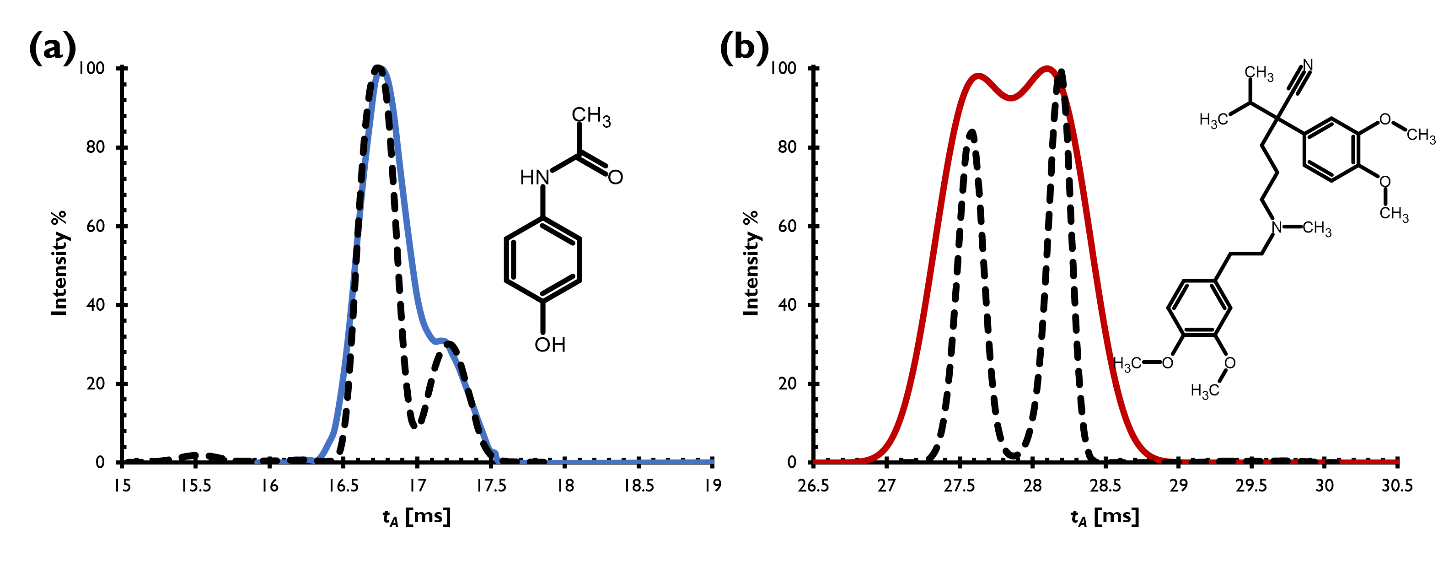


**Figure S1.** Experimental DTIM-MS arrival time spectra of [M+H]^+^ ions of (a) acetaminophen and (b) verapamil from Major Mix IMS/ToF calibration kit (CCS Major Mix), which are further resolved using high resolution demultiplexing (7) (black dashed line).

**Table S1.** Waters Major Mix IMS/ToF calibration kit (CCS Major Mix) ions in positive ESI mode: ion species, mass to charge ratio and used reference *CCS_N2,ref_* (Waters) as well as new single-field *^DT^CCS_N2_* reference values (average; n=3) and *^DT^CCS_N2_* from the CCS Compendium(8); ions observed to exhibit non-uniform arrival time distribution with DTIM-MS are indicated with an asterisk*.

| ***Waters Major Mix IMS/ToF calibration Kit; ESI pos.*** | | | | | ***CCS Compendium***(8) | | ***BOKU (single-field, n=3)*** | | | |
| --- | --- | --- | --- | --- | --- | --- | --- | --- | --- | --- |
| **Compound** | **Formula** | **Ion species** | **m/z** | ***CCS_N2,ref_* [Å²]** | ***^DT^CCS_N2_* [Å²]** | **Bias** | ***^DT^CCS_N2_* [Å²]** | **SD [Å²]** | **RSD** | **Bias** |
| *Acetaminophen | C8H9NO2 | [M+H]+ | 152.0706 | 130.4 | 131.4 | -0.8% | 132.81 | 0.22 | 0.2% | -1.8% |
| Caffeine | C8H10N4O2 | [M+H]^+^ | 195.0877 | 138.2 | 140.2 | -1.4% | 140.67 | 0.31 | 0.2% | -1.8% |
| Sulfaguanidine | C7H10N4O2S | [M+H]^+^ | 215.0597 | 146.8 | 148.4 | -1.1% | 148.76 | 0.16 | 0.1% | -1.3% |
| Sulfadimethoxine | C12H14N4O4S | [M+H]^+^ | 311.0809 | 168.4 | 169.4 | -0.6% | 169.52 | 0.07 | <0.1% | -0.7% |
| L-Val-Tyr-Val | C19H29N3O5 | [M+H]^+^ | 380.2180 | 191.7 | 191.3 | 0.2% | 192.98 | 0.22 | 0.1% | -0.7% |
| *Verapamil | C27H38N2O4 | [M+H]^+^ | 455.2904 | 208.8 | 205.4 | 1.7% | 210.28 | 0.12 | 0.1% | -0.7% |
| Terfenadine | C32H41NO2 | [M+H]^+^ | 472.3210 | 228.7 | 227.0 | 0.7% | 228.77 | 0.22 | 0.1% | <0.1% |
| Leucine-enkephalin | C28H37N5O7 | [M+H]^+^ | 556.2766 | 229.8 | 228.8 | 0.4% | 230.48 | 0.39 | 0.2% | -0.3% |
| Reserpine | C33H40N2O9 | [M+H]^+^ | 609.2807 | 252.3 | 250.3 | 0.8% | 251.64 | 0.23 | 0.1% | 0.3% |
| Polyalanine n=7 | C21H37N7O8 | [M+H]^+^ | 516.2776 | 211.0 | 209.7 | 0.6% | 209.95 | 0.18 | 0.1% | 0.5% |
| Polyalanine n=8 | C24H42N8O9 | [M+H]^+^ | 587.3148 | 228.0 | 226.2 | 0.8% | 226.24 | 0.70 | 0.3% | 0.8% |
| Polyalanine n=9 | C27H47N9O10 | [M+H]^+^ | 658.3519 | 243.0 | 239.9 | 1.3% | 239.95 | 0.17 | 0.1% | 1.3% |
| Polyalanine n=10 | C30H52N10O11 | [M+H]^+^ | 729.3890 | 256.0 | 252.5 | 1.4% | 251.88 | 0.66 | 0.3% | 1.6% |
| Polyalanine n=11 | C33H57N11O12 | [M+H]^+^ | 800.4261 | 271.0 | 265.7 | 2.0% | 265.42 | 0.84 | 0.3% | 2.1% |
| Polyalanine n=12 | C36H62N12O13 | [M+H]^+^ | 871.4632 | 282.0 | 278.5 | 1.3% | 278.19 | 0.65 | 0.2% | 1.4% |
| Polyalanine n=13 | C39H67N13O14 | [M+H]^+^ | 942.5003 | 294.0 | 290.8 | 1.1% | 290.92 | 0.68 | 0.2% | 1.1% |

**Table S2.** Waters Major Mix IMS/ToF calibration kit ions (CCS Major Mix) in negative ESI mode: ion species, mass to charge ratio and used reference *CCS_N2,ref_* (Waters) as well as new single-field *^DT^CCS_N2_* values (average; n=3) and *^DT^CCS_N2_* values from the CCS Compendium(8); ions observed to exhibit non-uniform arrival time distribution with DTIM-MS are indicated with an asterisk*.

| ***Waters Major Mix IMS/ToF calibration Kit; ESI neg.*** | | | | | ***CCS Compendium***(8) | | ***BOKU (single-field, n=3)*** | | | |
| --- | --- | --- | --- | --- | --- | --- | --- | --- | --- | --- |
| **Compound** | **Formula** | **Ion** | ***m/z*** | ***CCS_N2,ref_* [Å²]** | ***^DT^CCS_N2_* [Å²]** | **Bias** | ***^DT^CCS_N2_* [Å²]** | **SD [Å²]** | **RSD** | **Bias** |
| Acetaminophen | C8H9NO2 | [M-H]^-^ | 150.0561 | 131.5 | 132.7 | -0.9% | 133.71 | 1.04 | 0.8% | -1.7% |
| Sulfaguanidine | C7H10N4O2S | [M-H]^-^ | 213.0452 | 145.2 | 146.6 | -1.0% | 148.45 | 1.16 | 0.8% | -2.2% |
| Sulfadimethoxine | C12H14N4O4S | [M-H]^-^ | 309.0663 | 170.1 | 171.3 | -0.7% | 172.3 | 0.28 | 0.2% | -1.3% |
| *L-Val-Tyr-Val | C19H29N3O5 | [M-H]^-^ | 378.2034 | 192.5 | 185.2 | 3.9% | 193.71 | 0.44 | 0.2% | -0.6% |
| Leucine-enkephalin | C28H37N5O7 | [M-H]^-^ | 554.2620 | 225.3 | 222.6 | 1.2% | 222.34 | 0.19 | 0.1% | 1.3% |
| Reserpine | C33H40N2O9 | [M-H]^-^ | 607.2661 | 265.2 | n.d. | n.d. | 262.22 | 1.63 | 0.6% | 1.1% |
| Polyalanine n=9 | C27H47N9O10 | [M-H]^-^ | 656.3373 | 242.1 | 238.4 | 1.6% | 238.75 | 0.38 | 0.2% | 1.4% |
| Polyalanine n=10 | C30H52N10O11 | [M-H]^-^ | 727.3744 | 255.9 | 251.9 | 1.6% | 251.77 | 0.05 | 0.0% | 1.6% |
| Polyalanine n=11 | C33H57N11O12 | [M-H]^-^ | 798.4115 | 268.5 | 264.3 | 1.6% | 264.62 | 0.62 | 0.2% | 1.5% |
| Polyalanine n=12 | C36H62N12O13 | [M-H]^-^ | 869.4487 | 280.2 | 275.9 | 1.6% | 276.58 | 1.16 | 0.4% | 1.3% |
| Polyalanine n=13 | C39H67N13O14 | [M-H]^-^ | 940.4858 | 294.6 | 289.9 | 1.6% | 289.17 | 0.60 | 0.2% | 1.9% |

**Table S3.** Single-field *^DT^CCS_N2_* reference values (average; n=3) of steroid ions used as an external calibrant mixture for *^TW^CCS_N2_* calibration in this work (ST) (2).

| **Commercial name** | **Ion** | ***m/z*** | ***^DT^CCS_N2_* (Å^2^)**(2) |
| --- | --- | --- | --- |
| Trenbolone | [M+H]^+^ | 271.1693 | 166.70 |
| Boldenone | [M+H]^+^ | 287.2006 | 170.80 |
| Epi-19-nortestosterone | [M+H]^+^ | 275.2006 | 169.94 |
| Progesterone | [M+H]^+^ | 315.2319 | 181.38 |
| 17-Hydroxyprogesterone | [M+H]^+^ | 331.2268 | 183.60 |
| Cortisone | [M+H]^+^ | 361.2010 | 188.79 |
| Dexamethasone | [M+H]^+^ | 393.2072 | 191.20 |
| Testosterone benzoate | [M+H]^+^ | 393.2424 | 204.83 |
| Desonide | [M+H]^+^ | 417.2272 | 200.65 |
| 17-Caproxyprogesterone | [M+H]^+^ | 429.2999 | 215.30 |
| Boldenone undecylenate | [M+H]^+^ | 453.3363 | 222.39 |
| Testosterone glucuronide | [M+H]^+^ | 465.2483 | 221.25 |
| Triamcinolone 16,21-diacetate | [M+H]^+^ | 479.2076 | 211.84 |
| Estrone 3-sulfate | [M-H]^-^ | 349.1104 | 191.66 |
| 17α-Estradiol 3-sulfate | [M-H]^-^ | 351.1261 | 192.69 |
| Boldenone sulfate | [M-H]^-^ | 365.1400 | 191.03 |
| DHEA sulfate/Prasterone sulfate | [M-H]^-^ | 367.1574 | 196.81 |
| Epiandrosterone sulfate | [M-H]^-^ | 369.1730 | 197.73 |
| Estradiol 3-glucuronide | [M-H]^-^ | 447.2013 | 220.45 |
| 19-Noretiocholanolone glucuronide | [M-H]^-^ | 451.2326 | 204.71 |
| Testosterone glucuronide | [M-H]^-^ | 463.2326 | 219.47 |

**Table S4.** Single-field *^DT^CCS_N2_* values (average; n=3) determined for SIL-steroids used for internal correction of *^TW^CCS_N2_* in this work.

| **Name** | **Formula** | **Ion** | ***m/z*** | ***^DT^CCS_N2_***  **[A²]** | **SD**  **[A²]** | **RSD**  **%** |
| --- | --- | --- | --- | --- | --- | --- |
| Trenbolone d3 | C_18_H_19_^2^H_3_O_2_ | [M+H]^+^ | 274.19 | 165.60 | <0.01 | <0.1% |
| Trenbolone d3 | C_18_H_19_^2^H_3_O_2_ | [M+Na]^+^ | 296.17 | 192.42 | 0.05 | <0.1% |
| Methyltestosterone d3 | C_20_H_27_^2^H_3_O_2_ | [M+H]^+^ | 306.25 | 177.05 | 0.08 | <0.1% |
| Methyltestosterone d3 | C_20_H_27_^2^H_3_O_2_ | [M+Na]^+^ | 328.23 | 202.48 | 0.05 | <0.1% |
| Prednisolone d6 | C_21_H_22_^2^H_6_O_5_ | [M+H]^+^ | 367.24 | 185.48 | 0.10 | 0.1% |
| Prednisolone d6 | C_21_H_22_^2^H_6_O_5_ | [M+Na]^+^ | 389.22 | 211.95 | 0.01 | <0.1% |
| Boldenone sulfate d3 | C_19_H_23_^2^H_3_O_5_S | [M+H]^+^ | 370.18 | 189.28 | 0.16 | 0.1% |
| Boldenone sulfate d3 | C_19_H_23_^2^H_3_O_5_S | [M+Na]^+^ | 392.16 | 214.48 | 0.26 | 0.1% |
| Dexamethasone d4 | C_22_H_25_^2^H_4_FO_5_ | [M+H]^+^ | 397.23 | 189.96 | 0.30 | 0.2% |
| Dexamethasone d4 | C_22_H_25_^2^H_4_FO_5_ | [M+Na]^+^ | 419.21 | 216.56 | 0.07 | <0.1% |
| Epitestosterone glucuronate d3 | C_25_H_33_^2^H_3_O_8_ | [M+H]^+^ | 468.27 | 201.46 | 0.70 | 0.3% |
| Epitestosterone glucuronate d3 | C_25_H_33_^2^H_3_O_8_ | [M+Na]^+^ | 490.25 | 210.48 | 0.12 | 0.1% |
| Testosterone (2,3,4-^13^C) | C_16_^13^C_3_H_28_O_2_ | [M+H]^+^ | 292.23 | 172.41 | 0.06 | <0.1% |
| Testosterone (2,3,4-^13^C) | C_16_^13^C_3_H_28_O_2_ | [M+Na]^+^ | 314.21 | 197.65 | 0.13 | 0.1% |
| Estradiol (3,4-^13^C) | C_16_^13^C_2_H_24_O_2_ | [M+H]^+^ | 275.19 | 165.55 | 0.07 | <0.1% |
| Estradiol (3,4-^13^C) | C_16_^13^C_2_H_24_O_2_ | [M+Na]^+^ | 297.17 | 192.46 | 0.06 | <0.1% |
| Progesterone d9 | C_21_H_21_^2^H_9_O_2_ | [M+H]^+^ | 324.29 | 180.79 | 0.09 | <0.1% |
| Progesterone d9 | C_21_H_21_^2^H_9_O_2_ | [M+Na]^+^ | 346.27 | 206.09 | 0.03 | <0.1% |
| Testosterone benzoate d3 | C_26_H_29_^2^H_3_O_3_ | [M+H]^+^ | 396.26 | 207.67 | 0.05 | <0.1% |
| Testosterone benzoate d3 | C_26_H_29_^2^H_3_O_3_ | [M+Na]^+^ | 418.24 | 233.34 | 0.02 | <0.1% |
| Boldenone sulfate d3 | C_19_H_23_^2^H_3_O_5_S | [M-H]^-^ | 368.16 | 191.73 | 0.15 | 0.1% |
| Epitestosterone glucuronate d3 | C_25_H_33_^2^H_3_O_8_ | [M-H]^-^ | 466.25 | 219.92 | 0.54 | 0.2% |


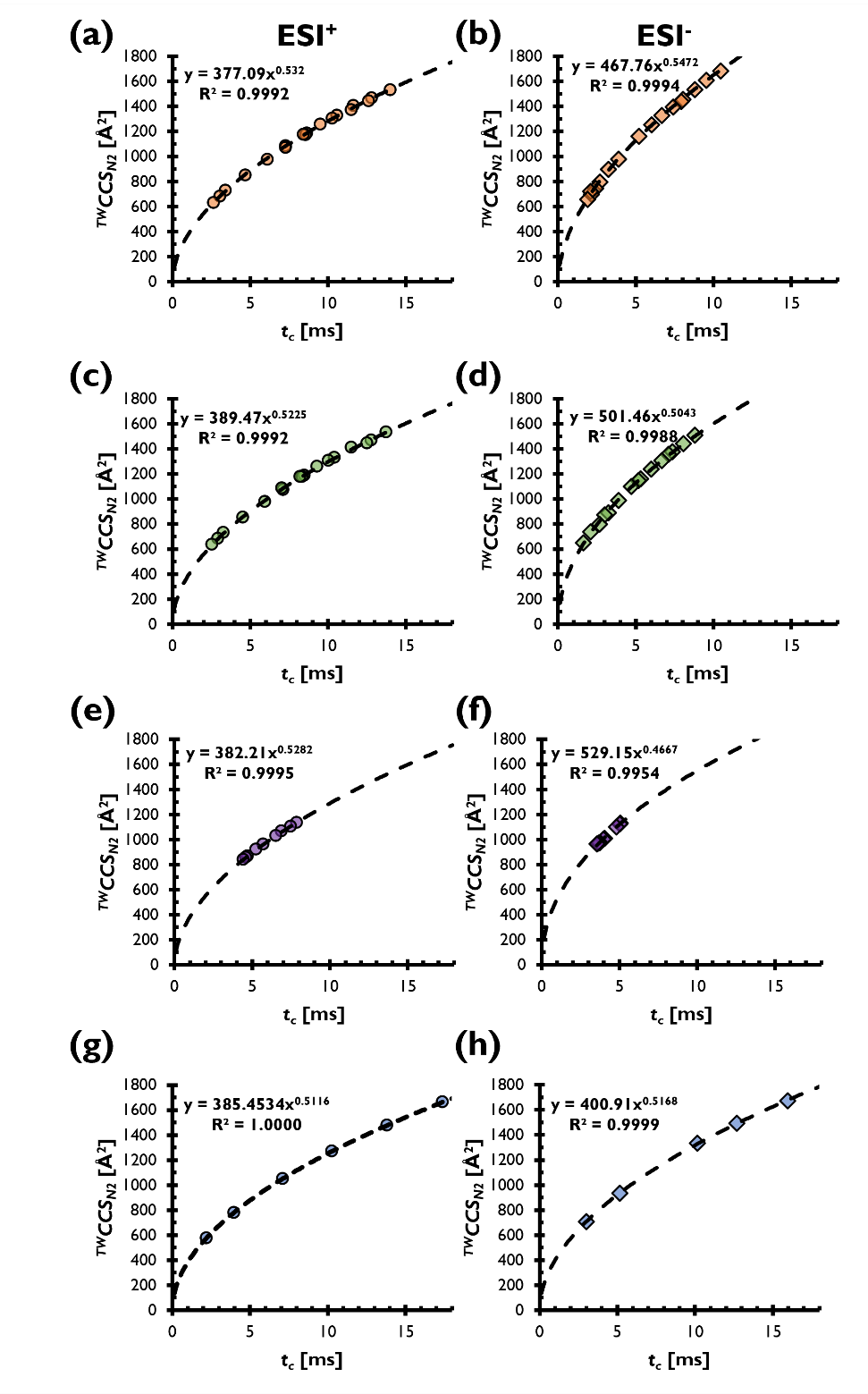


**Figure S2.**  Examples for applied calibration functions (left panels) in ESI^+^ mode and (right panels) in ESI^-^ mode using Waters CCS Major Mix and default reference values (a-b), Waters CCS Major Mix and new *^D^CCS_N2_* reference values (c-d), steroid calibrant ions with new *^DT^CCS_N2_* reference values (e-f) and Agilent ESI-L tune mix ions (g-h).


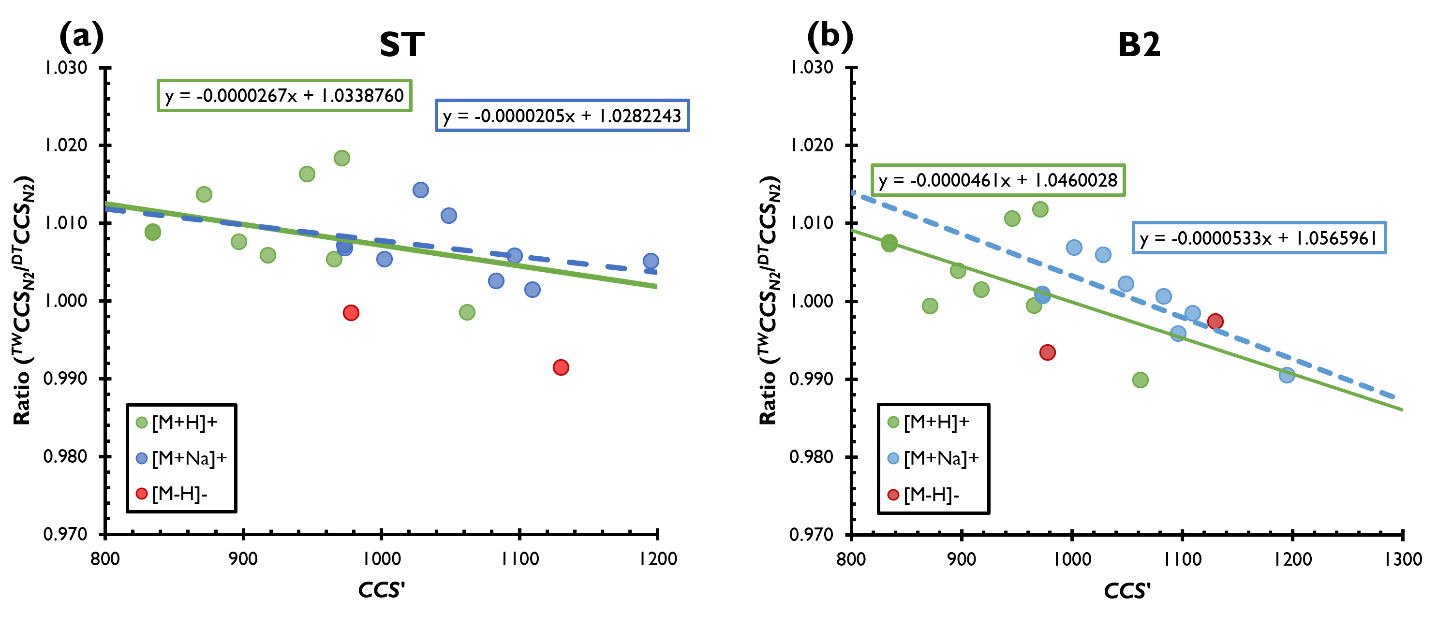


**Figure S3.** Ratios of measured ^TW^CCS_N2_ values and reference ^DT^CCS_N2_ with respect to CCS’ plotted for SIL-steroids using linear models applied to (a) B2 dataset and (b) ST dataset. Models for internal standards (ISTDs) were used to derive a correction function to be applied to correct externally calibrated ^TW^CCS_N2_ of analyte molecules as a function of CCS’.

Individual correction factors were calculated based on ions’ *CCS’* linear models derived from SIL-ISTD information were used to calculate correction factors. Results for internal correction are presented in detail in **Figure S4**.


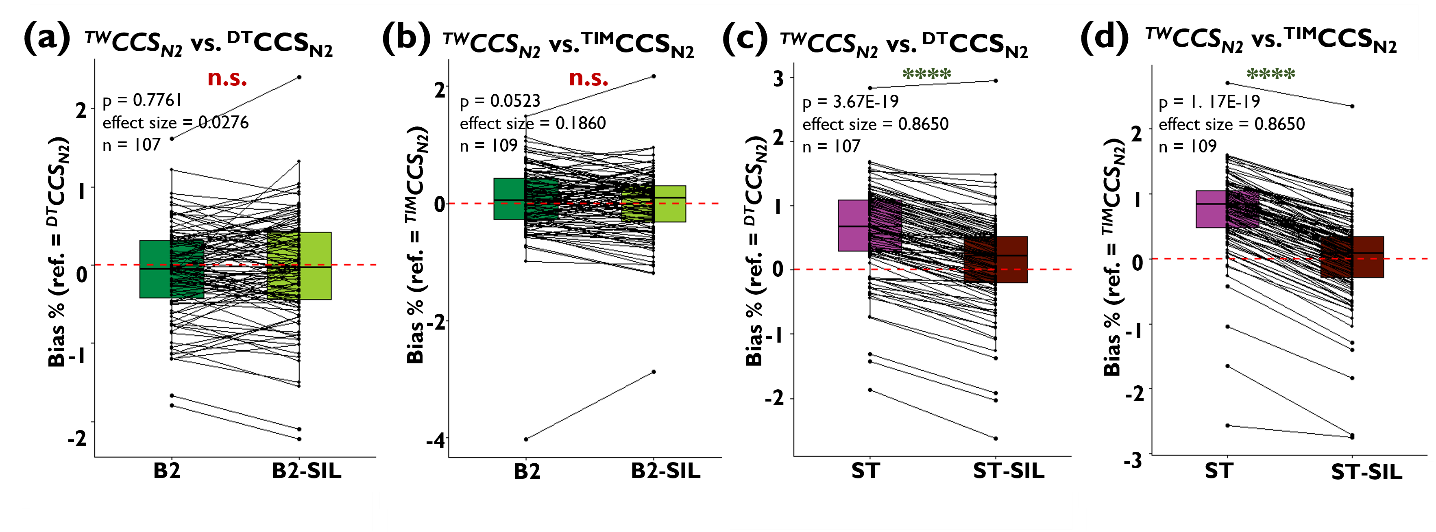


**Figure S4.** Effect of a linear SIL-based internal correction on bias distribution for dataset B2 using (a) *^DT^CCS_N2_* and (b) *^TIM^CCS_N2_* as reference, and for dataset ST using (c) *^DT^CCS_N2_* and (d) *^TIM^CCS_N2_* as reference. Significance and effect size were calculated using a Wilcoxon test.

*
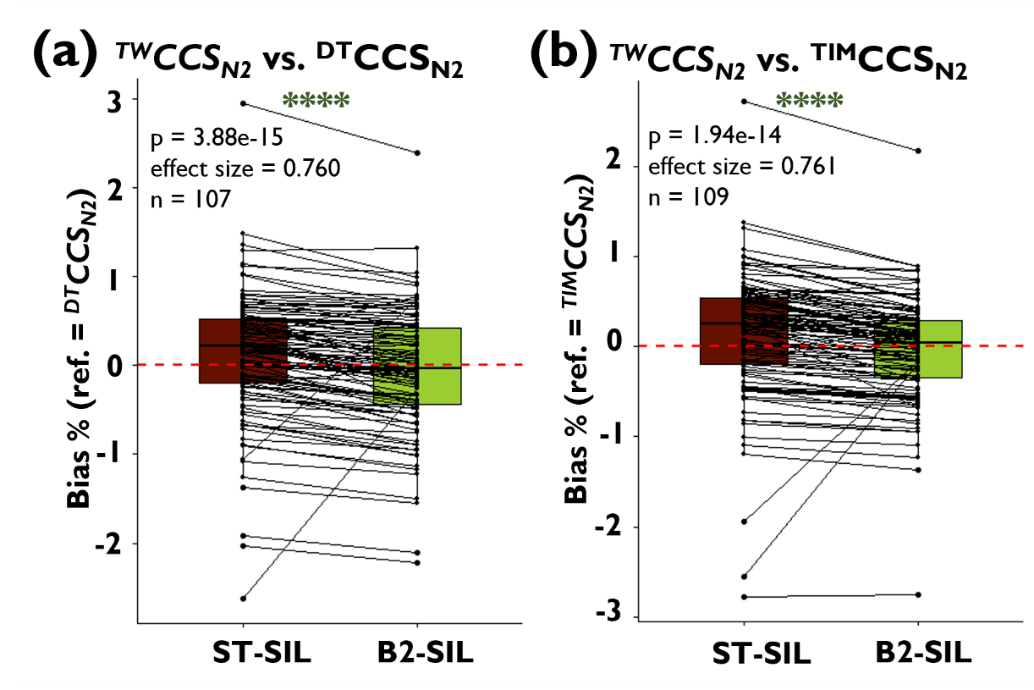
*

**Figure S5.** Bias (%) distribution of internally corrected datasets ST-SIL and B2-SIL using (a) *^DT^CCS_N2_* or (b) *^TIM^CCS_N2_* as reference. Significance (*p* values) and effect size were calculated using a Wilcoxon test.

## Calculated Ion Structures and Predicted *CCS_N2_* Values

| 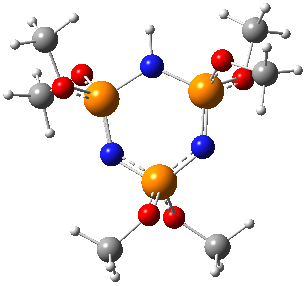  [HMP+H]^+^-a  19.1 | 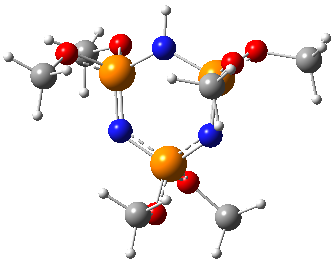  [HMP+H]^+^-b  0.0 | 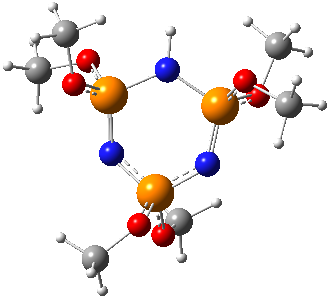  [HMP+H]^+^-c  17.9 |
| --- | --- | --- |
| 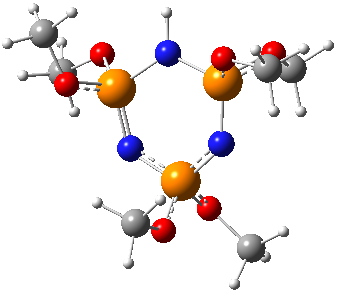  [HMP+H]^+^-d  4.7 | 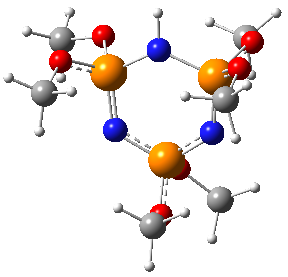  [HMP+H]^+^-e  3.9 | 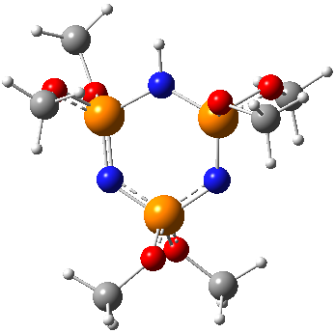  [HMP+H]^+^-f  3.8 |

**Figure S6**. The wB97xD-optimized structures and relative Gibbs free energies of six conformers of protonated hexamethoxyphosphazene (HMP) in gas phase and 298.15 K. The energies are in kJ mol^-1^.

**Table S5**. Comparison of the experimental *^DT^CCS_N2_* and theoretical *CCS_N2_* data of the hexamethoxyphosphazene, [HMP+H]^+^, optimized by ωB97xD and B3LYP methods.

|  | **exp** | **ωB97xD** | | **B3LYP** | |
| --- | --- | --- | --- | --- | --- |
| **Ion** | **^DT^CCS_N2_** | **CCS_N2_ (Å^2^)** | **Error%** | **CCS_N2_ (Å^2^)** | **Error%** |
| [HMP+H]^+^-a | 153.7 | 168.7 | 9.76 | 169.4 | 10.21 |
| [HMP+H]^+^-b |  | 158.9 | 3.38 | 161.3 | 4.94 |
| [HMP+H]^+^-c |  | 165.4 | 7.61 | 166.5 | 8.32 |
| [HMP+H]^+^-d |  | 161.1 | 4.81 | 164.9 | 7.28 |
| [HMP+H]^+^-e |  | 153.3 | -0.26 | 156.0 | 1.49 |
| [HMP+H]^+^-f |  | 161.3 | 4.94 | 163.9 | 6.63 |

| 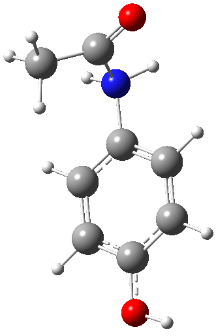  [Ac+H]^+^-a  50.8 | 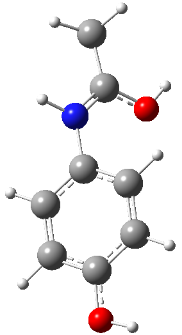  [Ac+H]^+^-b  0.0 | 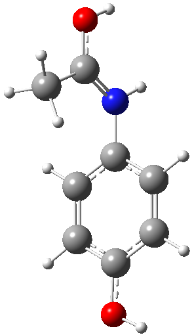  [Ac+H]^+^-c  5.2 | 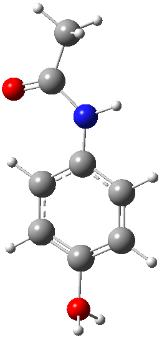  [Ac+H]^+^-d  128.0 |
| --- | --- | --- | --- |

**Figure S7**. The wB97xD-optimized structures and relative Gibbs free energies of protomers of acetaminophen (Ac) in gas phase and 298.15 K. The energies are in kJ mol^-1^.

**Table S6**. Comparison of the experimental *^DT^CCS_N2_* and theoretical *CCS_N2_* data of acetaminophen, [Ac+H]^+^, optimized by ωB97xD and B3LYP methods.

|  | **exp** | **ωB97xD** | | **B3LYP** | |
| --- | --- | --- | --- | --- | --- |
| **Ion** | **^DT^CCS_N2_** | **CCS_N2_ (Å^2^)** | **Error%** | **CCS_N2_ (Å^2^)** | **Error%** |
| [Ac+H]^+^-a | 131.96 | 132.5 | 0.41 | 131.4 | -0.42 |
| [Ac+H]^+^-b |  | 131.5 | -0.35 | 127.6 | -3.30 |
| [Ac+H]^+^-c |  | 131.3 | -0.50 | 129.7 | -1.71 |
| [Ac+H]^+^-d |  | 139.9 | 6.02 | 138.3 | 4.80 |

| 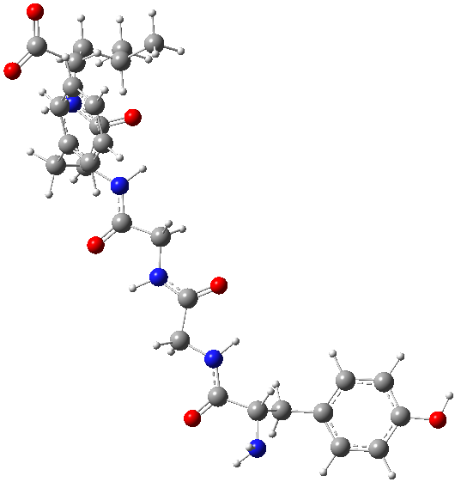  [Leu-H]^-^-a  67.6 | 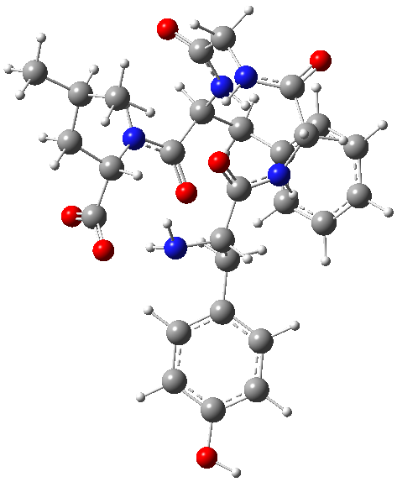  [Leu-H]^-^-b  110.5 | 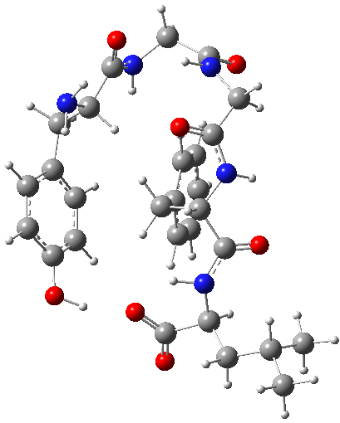  [Leu-H]^-^-c  37.2 |
| --- | --- | --- |
| 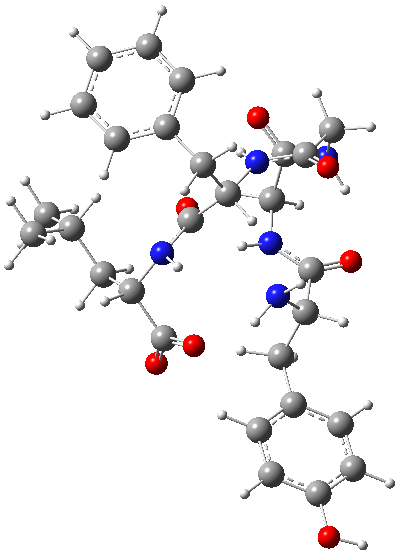  [Leu-H]^-^-d  67.4 | 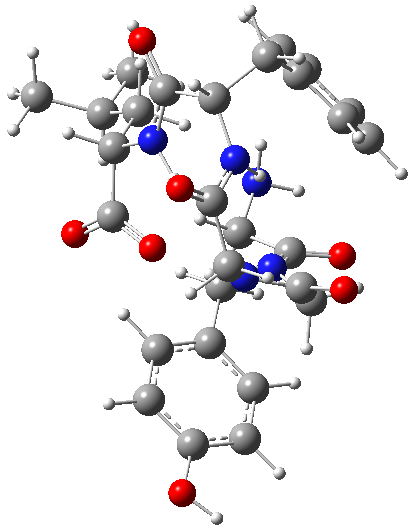  [Leu-H]^-^-e  0.0 | 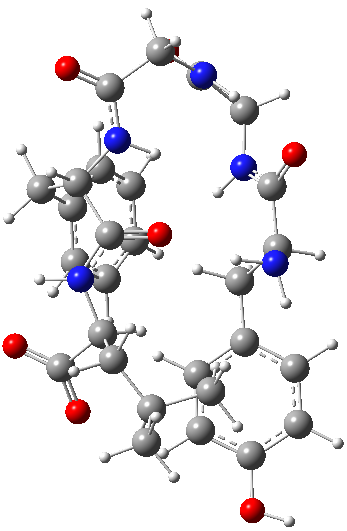  [Leu-H]^-^-f  128.0 |

**Figure S8**. The wB97xD-optimized structures and relative Gibbs free energies of conformers of deprotonated leucine enkephalin (Leu) in gas phase and 298.15 K. The energies are in kJ mol^-1^.

**Table S7**. Comparison of the experimental *^DT^CCS_N2_* and theoretical *CCS_N2_* data of the deprotomers of leucine enkephalin, [Leu-H]^-^, optimized by ωB97xD and B3LYP methods.

|  | **exp** | **ωB97xD** | | | **B3LYP** | | |  |
| --- | --- | --- | --- | --- | --- | --- | --- | --- |
| **Ion** | **^DT^CCS_N2_** | **CCS_N2_ (Å^2^)** | | **Error%** | **CCS_N2_ (Å^2^)** | | **Error%** |  |
| [Leu-H]^-^-a | 221.8 | 288.8 | | 30.21 | 292.6 | | 31.9 |  |
| [Leu-H]^-^-b |  | 234.7 | | 5.81 | 242.9 | | 9.5 |  |
| [Leu-H]^-^-c |  | 234.8 | | 5.86 | 250.0 | | 12.7 |  |
| [Leu-H]^-^-d |  | 240.8 | | 8.56 | 255.7 | | 15.3 |  |
| [Leu-H]^-^-e |  | 228.0 | | 2.79 | 242.3 | | 9.2 |  |
| [Leu-H]^-^-f |  | 230.1 | | 3.74 | 240.9 | | 8.6 |  |
| 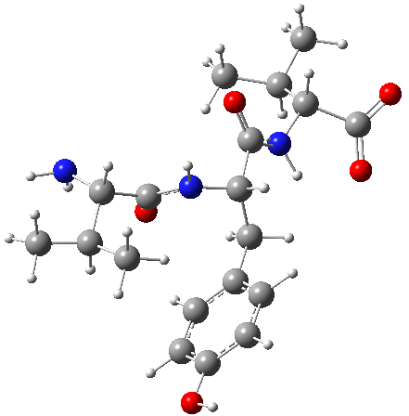  [VTV-H]^-^-a  51.2 | | | 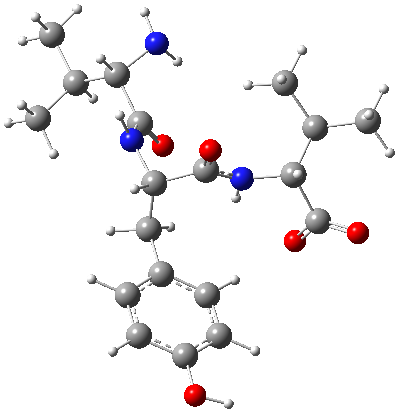  [VTV-H]^-^-b  48.1 | | | 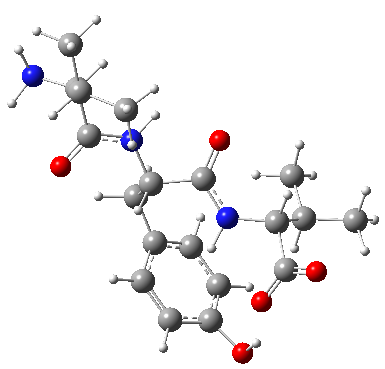  [VTV-H]^-^-c  7.1 | | |
| 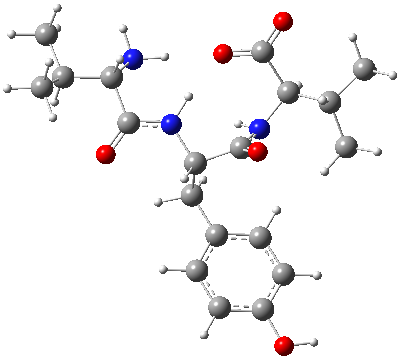  [VTV-H]^-^-d  40.0 | | | 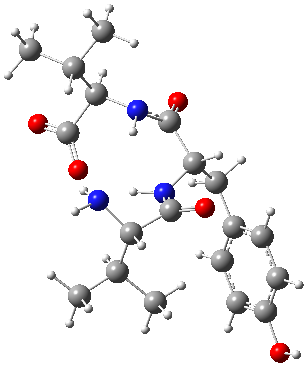  [VTV-H]^-^-e  8.4 | | | 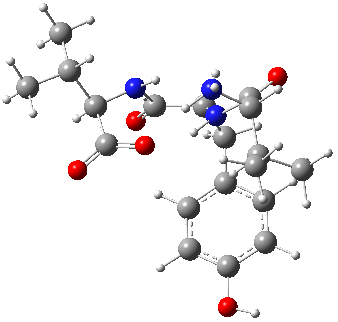  [VTV-H]^-^-f  0.0 | | |

**Figure S9** The wB97xD-optimized structures and relative Gibbs free energies of conformers of deprotonated L-Val-Tyr-Val (VTV) in gas phase and 298.15 K. The energies are in kJ mol^-1^.

**Table S8**. Comparison of the experimental *^DT^CCS_N2_* and theoretical *CCS_N2_* data of the deprotomers of deprotonated L-Val-Tyr-Val, [VTV-H]^-^, optimized by ωB97xD and B3LYP methods.

|  | **exp** | **ωB97xD** | | **B3LYP** | |
| --- | --- | --- | --- | --- | --- |
| **Ion** | **^DT^CCS_N2_** | **CCS_N2_ (Å^2^)** | **Error%** | **CCS_N2_ (Å^2^)** | **Error%** |
| [VTV-H]^-^-a | 193.1  (184.9) | 213.9 | 10.77 | 216.0 | 11.86 |
| [VTV-H]^-^-b |  | 212.5 | 10.04 | 213.6 | 10.62 |
| [VTV-H]^-^-c |  | 202.3 | 4.76 | 203.5 | 5.38 |
| [VTV-H]^-^-d |  | 207.9 | 7.66 | 210.1 | 8.80 |
| [VTV-H]^-^-e |  | 201.8 | 4.51 | 206.3 | 6.83 |
| [VTV-H]^-^-f |  | 197.4 | 2.23 | 202.2 | 4.71 |

| 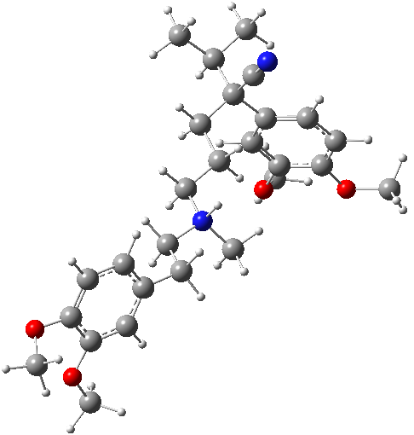  [VP+H]^+^-a  42.1 | 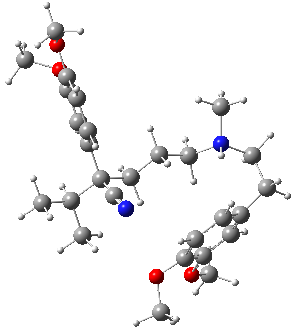  [VP+H]^+^-b  22.9 | 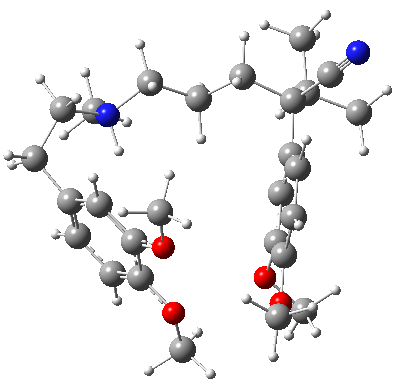  [VP+H]^+^-c  24.6 |
| --- | --- | --- |
| 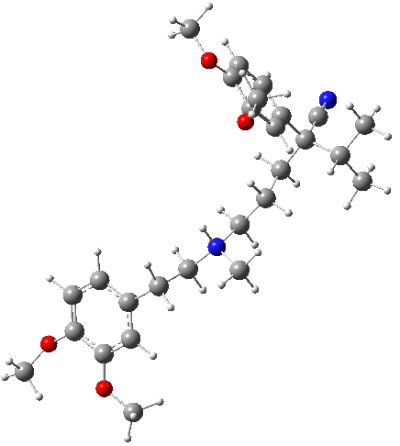  [VP+H]^+^-d  62.2 | 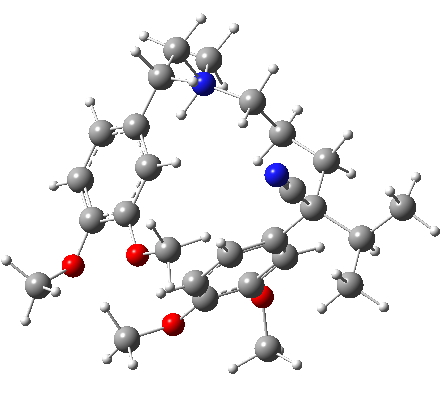  [VP+H]^+^-e  0.0 | 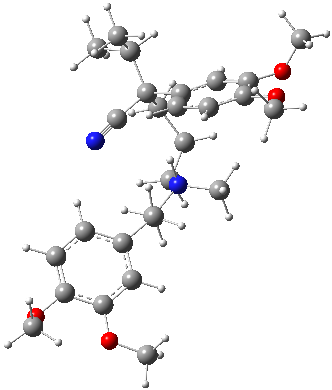  [VP+H]^+^-f  5.0 |

**Figure S10**. The wB97xD-optimized structures and relative Gibbs free energies of conformers of protonated verapamil (VP) in gas phase and 298.15 K. The energies are in kJ mol^-1^.

**Table S9**. Comparison of the experimental *^DT^CCS_N2_* and theoretical *CCS_N2_* data of the protonated verapamil, [VP+H]^+^, optimized by ωB97xD and B3LYP methods.

|  | **exp** | **ωB97xD** | | **B3LYP** | |
| --- | --- | --- | --- | --- | --- |
| **Ion** | **^DT^CCS_N2_** | **CCS_N2_ (Å^2^)** | **Error%** | **CCS_N2_ (Å^2^)** | **Error%** |
| [VP+H]^+^-a | 207.8  (212.1) | 241.7 | 16.31 | 245.5 | 18.14 |
| [VP+H]^+^-b |  | 223.1 | 7.36 | 232.4 | 11.83 |
| [VP-H]^+^-c |  | 209.9 | 1.01 | 219.5 | 5.63 |
| [VP+H]^+^-d |  | 245.4 | 18.1 | 245.4 | 18.09 |
| [VP+H]^+^-e |  | 211.2 | 1.63 | 218.0 | 4.91 |
| [VP+H]^+^-f |  | 223.5 | 7.55 | 234.7 | 12.94 |

| 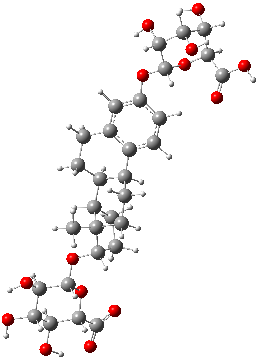  [ED-H]^-^-a  4.2 | 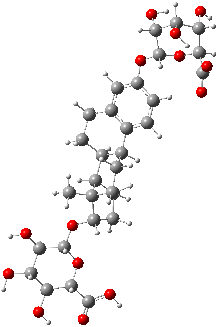  [ED-H]^-^-b  2.7 | 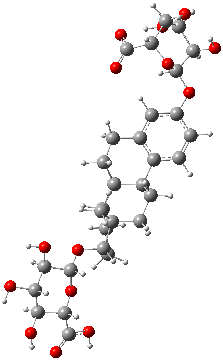  [ED-H]^-^-c  0.0 | 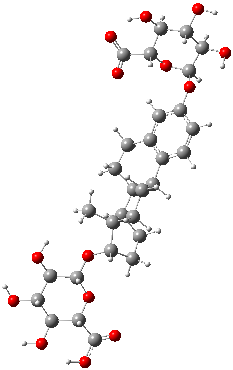  [ED-H]^-^-d  4.2 |
| --- | --- | --- | --- |
| 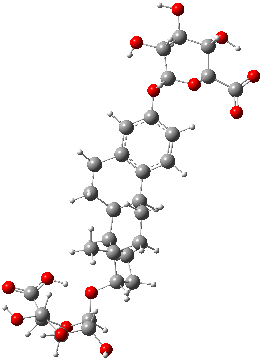  [ED-H]^-^-e  5.8 | 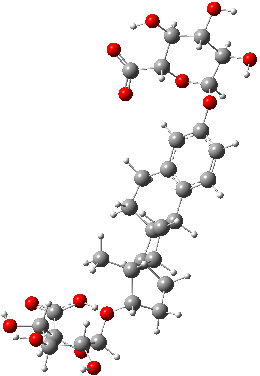  [ED-H]^-^-f  6.6 | 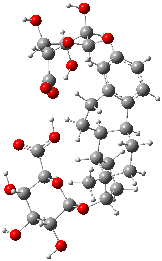  [ED-H]^-^-g  15.2 | 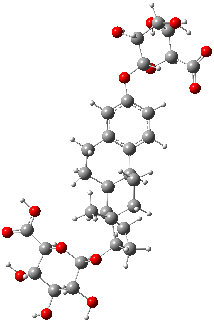  [ED-H]^-^-h  25.7 |

**Figure S11.** The wB97xD-optimized structures and relative Gibbs free energies of [ED-H]^-^ conformers in gas phase and 298.15 K. The energies are in kJ mol^-1^. The structures **a** and **b** are deprotomers and structures **c**, **d**, **e**, **f**, **g**, and **h** are conformers of the deprotomer **b**.

**Table S10**. The theoretical *CCS_N2_* data of the deprotonated ions of estradiol diglucuronide optimized by ωB97xD methods.

| **Ion** | **CCS_N2_ (Å^2^)** | |  |  |  |
| --- | --- | --- | --- | --- | --- |
| [ED-H]^-^-a | 283.8 | |  |  |  |
| [ED-H]^-^-b | 280.0 | |  |  |  |
| [ED-H]^-^-c | 279.2 | |  |  |  |
| [ED-H]^-^-d | 279.6 | |  |  |  |
| [ED-H]^-^-e | 271.9 | |  |  |  |
| [ED-H]^-^-f | 268.2 | |  |  |  |
| [ED-H]^-^-g | 241.0 | |  |  |  |
| [ED-H]^-^-h | 271.9 | |  |  |  |
| 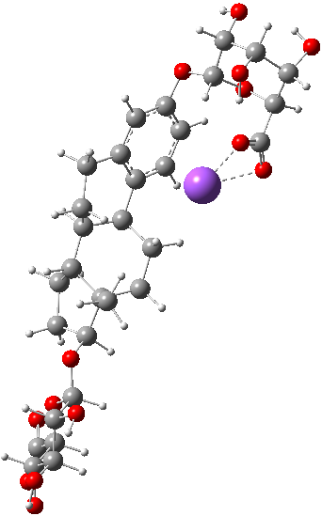  [ED+Na-2H]^-^-a  84.7 | | 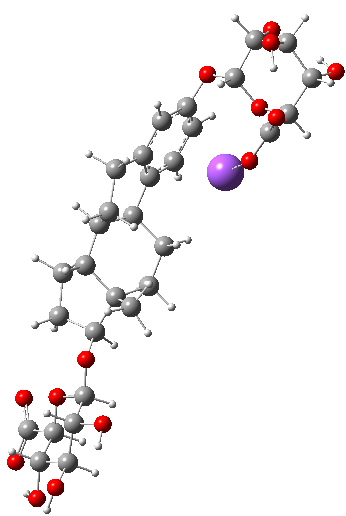  [ED+Na-2H]^-^-b  70.8 | | 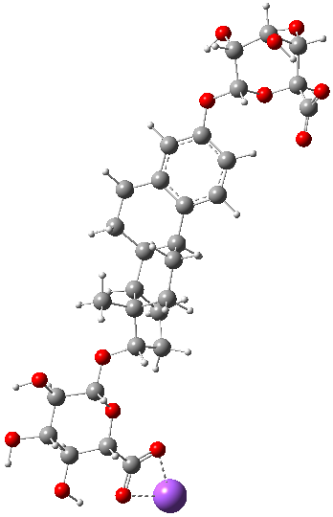  [ED+Na-2H]^-^-c  103.5 | 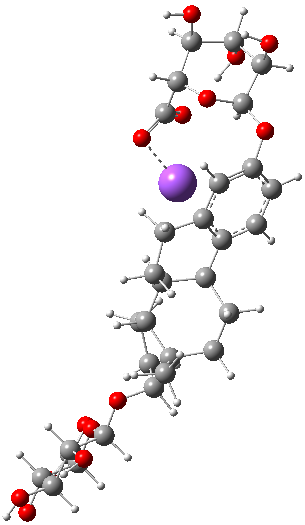  [ED+Na-2H]^-^-d  93.1 |
| 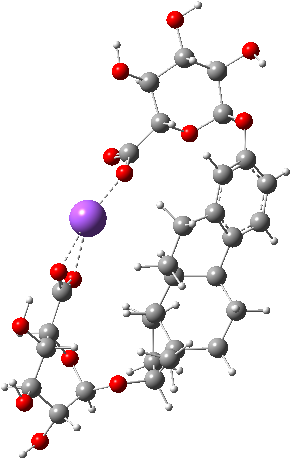  [ED+Na-2H]^-^-e  31.2 | | 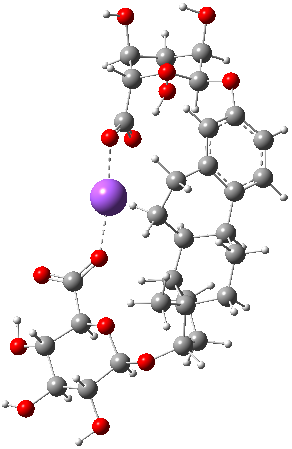  [ED+Na-2H]^-^-f  0.0 | | 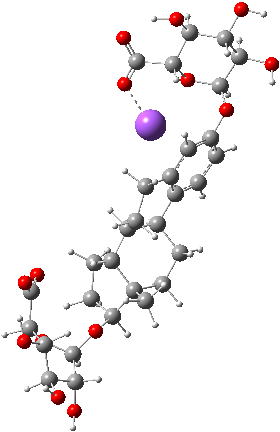  [ED+Na-2H]^-^-g  87.7 | 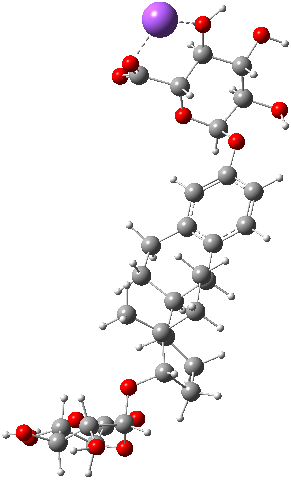  [ED+Na-2H]^-^-h  148.3 |

**Figure S12.** The wB97xD-optimized structures and relative Gibbs free energies of conformers of [ED+Na-2H]^-^ in gas phase and 298.15 K. The energies are in kJ mol^-1^.

**Table S11**. The theoretical *CCS_N2_* data of [ED+Na-2H]^-^ ions of estradiol diglucuronide optimized by ωB97xD methods.

| **Ion** | **CCS_N2_ (Å^2^)** |
| --- | --- |
| [ED+Na-2H]^-^-a | 281.7 |
| [ED+Na-2H]^-^-b | 287.1 |
| [ED+Na-2H]^-^-c | 299.8 |
| [ED+Na-2H]^-^-d | 280.1 |
| [ED+Na-2H]^-^-e | 249.1 |
| [ED+Na-2H]^-^-f | 252.3 |
| [ED+Na-2H]^-^-g | 271.9 |
| [ED+Na-2H]^-^-h | 288.1 |


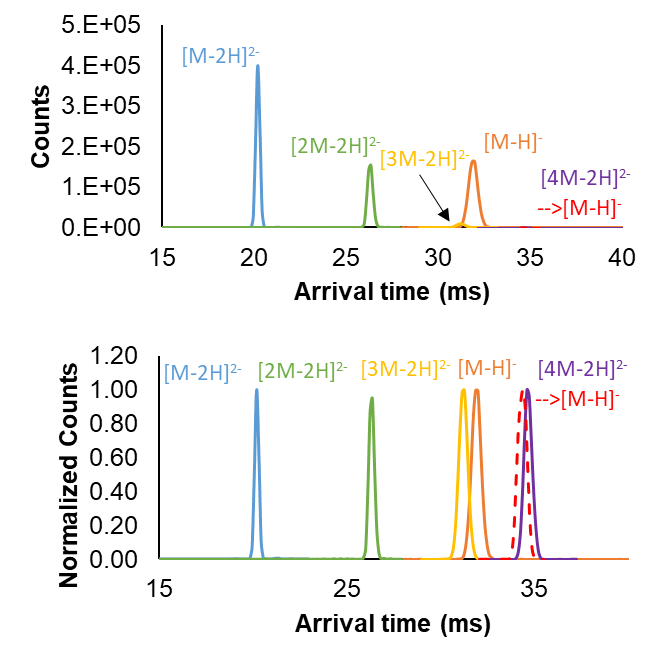


**Figure S13.** Experimental DTIM-MS data for estradiol diglucuronide in negative mode. The appearance of a post-IM fragment (red trace) matching [M-H]^-^ of a multimeric species (purple trace) using CID (20 V) with an arrival time closely matching the expected arrival time (~34 ms) according to TIM-MS and TWIM-MS results is highlighted.

**References**

1. Hernández-Mesa M, D’Atri V, Barknowitz G, Fanuel M, Pezzatti J, Dreolin N, et al. Interlaboratory and Interplatform Study of Steroids Collision Cross Section by Traveling Wave Ion Mobility Spectrometry. Anal Chem. 2020 Apr 7;92(7):5013–22.

2. Feuerstein ML, Hernández-Mesa M, Kiehne A, Le Bizec B, Hann S, Dervilly-Pinel G, et al. Comparability of Steroid Collision Cross Sections Using Three Different IM-HRMS Technologies: An Interplatform Study. ChemRxiv. 2022; 10.26434/chemrxiv-2022-87k68

3. Ruotolo BT, Benesch JLP, Sandercock AM, Hyung SJ, Robinson CV. Ion mobility–mass spectrometry analysis of large protein complexes. Nat Protoc. 2008 Jul 1;3(7):1139–52.

4. Campuzano IDG, Giles K. Historical, current and future developments of travelling wave ion mobility mass spectrometry: A personal perspective. TrAC Trends Anal Chem. 2019 Nov 1;120:115620.

5. Hinnenkamp V, Klein J, Meckelmann SW, Balsaa P, Schmidt TC, Schmitz OJ. Comparison of CCS Values Determined by Traveling Wave Ion Mobility Mass Spectrometry and Drift Tube Ion Mobility Mass Spectrometry. Anal Chem. 2018 Oktober;90(20):12042–50.

6. Shvartsburg AA, Smith RD. Fundamentals of Traveling Wave Ion Mobility Spectrometry. Anal Chem. 2008 Dec 15;80(24):9689–99.

7. May JC, Knochenmuss R, Fjeldsted JC, McLean JA. Resolution of Isomeric Mixtures in Ion Mobility Using a Combined Demultiplexing and Peak Deconvolution Technique. Anal Chem. 2020 Jul 21;92(14):9482–92.

8. Picache JA, Rose BS, Balinski A, Leaptrot KL, Sherrod SD, May JC, et al. Collision cross section compendium to annotate and predict multi-omic compound identities. Chem Sci. 2018 Nov 27;10(4):983–93.
